# Virtual planning for corrections of hemifacial microsomia

**DOI:** 10.1515/iss-2021-0010

**Published:** 2023-10-17

**Authors:** Adrian Sugar, Peter Evans, Scott Bartlett, Steven Key

**Affiliations:** Morriston Hospital, Swansea Bay University Health Board, Maxillofacial and Cleft Units, Swansea, UK; Department of Plastic Surgery, Childrens Hospital of Philadelphia, Philadelphia, PA, USA

**Keywords:** hemifacial microsomia, virtual surgical planning, facial reconstruction

## Abstract

**Objectives:**

This article describes the many anomalies within and outside the head and neck of hemifacial microsomia (HFM).

**Methods:**

The OMENS+ classification system is described with particular reference to the mandibular features classified by Pruzansky and modified by Kaban. The application of virtual surgical planning (VSP) to HFM, largely in children, is described and taken through to aspects indicated in maturity.

**Results:**

VSP is demonstrated with clinical cases examples in HFM patients for (1) grafts and flaps replacing missing parts of the zygomatic bone, temporo-mandibular joint and mandible, (2) distraction osteogenesis for lengthening of the mandibular ramus, advancement of the mandibular body, widening of the face and simultaneous mid-face and mandibular rotation, (3) implants and correction of microtia for bone anchored ear prostheses, (4) correction of microtia by autogenous ear construction, and (5) end stage rotational bimaxillary osteotomies.

**Conclusions:**

3D virtual and physical planning is a valuable adjunct to the treatment of this complex condition.

## Introduction

Hemifacial microsomia (HFM) is the most common craniofacial birth defect after cleft lip and palate (1 in 600 live births depending on country of origin and ethnicity) and craniosynostosis (approximately 1 in 2000 live births). HFM is essentially an abnormality of development of the embryonic first and second branchial arches but is also associated with many other congenital abnormalities in other parts of the body (Table 1) [1]. It is usually unilateral and always asymmetrical. It can be distinguished from Treacher Collins syndrome (TCS) which is also a syndrome of the first and second branchial arches but which is bilateral and generally symmetrical. TCS is inherited as an autosomal dominant gene but with approximately 65 % new mutations; the gene has been identified. The head and neck anomalies of HFM can sometimes be mistaken for torticollis and for plagiocephaly.

**Table 1:** Signs, symptoms and associated features found in Hemifacial Microsomia.

Patients with HFM may demonstrate aplasia, hypoplasia, reduced function, or malposition of the following:
– Mandible (especially the ramus) – Dental occlusion (which can be canted up on the affected side) – Teeth (abnormal formed, missing, ectopic and/or crowded) – Ear (microtia/anotia, accessory ear tags) – Hearing (the outer and/or middle ear may be missing or abnormally formed although the inner ear and the cochlea normally works well) – Conduction hearing loss, in 75 % of cases – Sensorineural hearing loss, in 11 % of cases – Maxilla – Malar/zygomatic bone – Temporal bone, especially the glenoid fossa – Frontal bone – Vertebrae/spine (40–60 % of cases) – Facial nerve (22 % of cases) – Macrostomia – Dermoid cysts (especially epibulbar) – Muscles (of mastication) – Fat – Temporal fascia – Parotid gland – Microphthalmia/anophthalmia – Colobomas – Lateral facial cleft – Rarely occipital encephalocele – Rarely developmental delay – Clefts of the lip and/or palate
Other systemic findings include congenital cardiac and other genetic abnormalities such as:
– Ventriculo-septal defect (VSD) – Patent ductus arteriosus – Fallot’s tetralogy (VSD, pulmonary stenosis, right ventricular hypertrophy and overriding aorta) – Tracheo-oesophageal fistula (TOF) as well as renal/ureteric abnormalities

**Figure 1:**
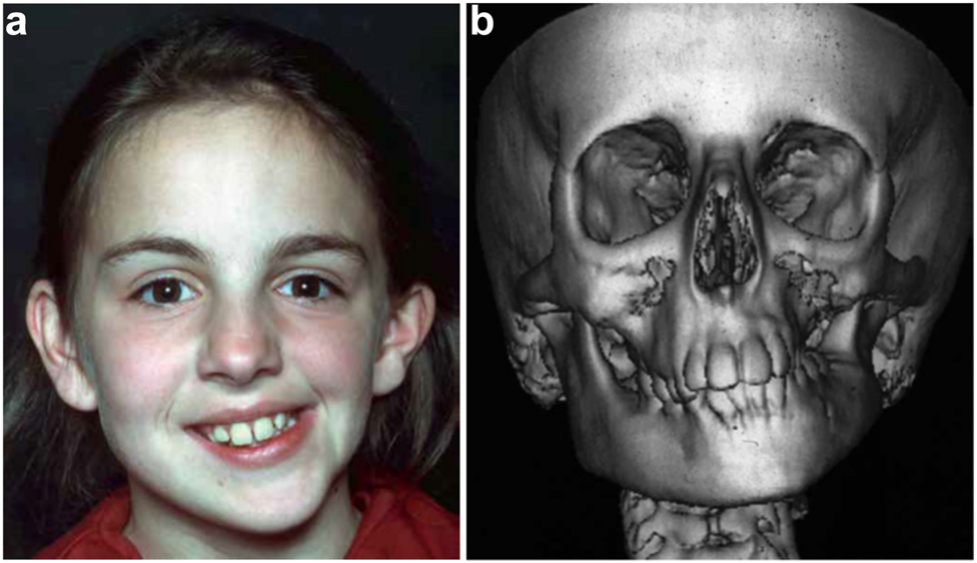
Clinical and CT example of Class II classification of HFM. (a) and (b) Example of Pruzansky IIa HFM aged 12 yrs.

**Figure 2:**
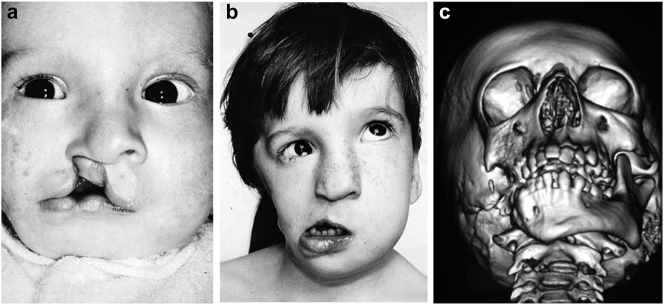
Clinical and CT examples of Class III classification of HFM. (a) Baby with Pruzansky III HFM. (b) Same child 10 yrs later. (c) 3DCT of an example of Pruzansky III HFM.

Hemifacial microsomia occurs in approximately 1:5,000 live births and displays a wide spectrum of abnormalities (Figures 1 and 2). Synonyms that are also in common usage are craniofacial microsomia, oculo-auriculo-vertebral-dysplasia and facial-oculo-auriculo-vertebral dysplasia. Goldenhar syndrome is a much overused term liked by paediatricians but only really refers to a small subgroup of patients with HFM who have significant spinal anomalies and epibulbar dermoid cysts.

## Aetiology

The aetiology of HFM is not fully understood. There does not appear to be a clearly inherited gene. Poswillo [2, 3] showed in animal experiments on mice and monkeys that a similar condition could be induced by creating a haematoma in the anastomosis precursor of the embryonic stapedial artery system. Similar but bilateral examples have also occurred following the use of retinoids in humans during pregnancy (e.g. thalidomide) and this has been repeated experimentally by Jacobsson [4, 5] in further animal experiments on rats.

## Features and growth

It is not clear whether the facial deformity of HFM gets worse with age and growth or not. A longitudinal study of the mandible by Kaban in 1984 [6] suggested that the deformity gets worse with age, and a further study by Polley in 1997 [7] suggested that the deformity did not get worse with growth. Our patient below (Figure 2a and b) shows significant worsening of the deformity with growth and we have observed considerable variability in development across our cohort of more than 100 patients.

In the neo-nate, the early identification of respiratory, cardiac, hearing, visual, cleft and spinal anomalies is important as their treatment may need to be prioritised. The definitive diagnosis and classification of HFM is largely produced after thorough clinical examination and high definition CT scan including 3D reformats [8, 9].

## Classification

Horgan et al. [10] offers the most comprehensive classification system as OMENS+.

O=Orbit
M=Mandible
E=Ear
N=Nerves
S=Soft tissues
+=Extracraniofacial features

The Mandible part of this classification uses the Kaban modification of the Pruzansky [11] classification (M2A and M2B, Pruzansky IIa and IIb) and overall these classifications are strong indicators for treatment.

I a small ramus with identifiable anatomy
II a functioning TMJ but with an abnormal shape and glenoid fossa (IIa and IIb as modified by Kaban [12])
  IIa the glenoid fossa is in an acceptable functional position
  IIb the TMJ is abnormally placed and cannot be incorporated in the surgical construction
III an absent ramus and non-existent glenoid fossa

Throughout this text going forwards, we will now refer to the Pruzansky classification as modified by Kaban as PK.

**Figure 3:**
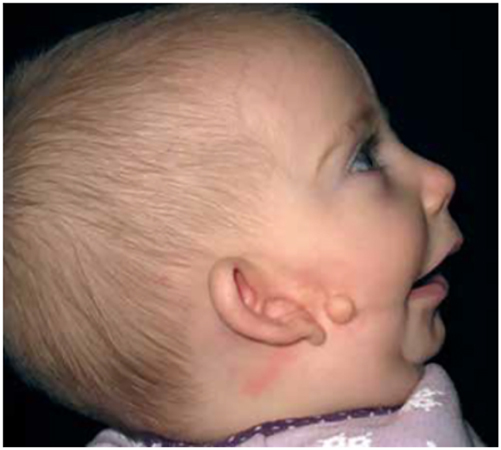
Clinical example of an incomplete Tessier no. 7 facial cleft.

HFM often shows its most severe manifestation as an incomplete Tessier no. 7 facial cleft [13] (Figure 3), with macrostomia, microtia, a pre-auricular ear tag and mandibular deformity along the line of the facial cleft.

The key issues for clinicians are whether to offer interventions and when. These will principally be determined by the wishes of the patient who will often be a child and their parents. From the clinical side, the severity of the condition and whether it is becoming progressively worse will be of major importance.

## Virtual planning of treatment of HFM

This paper will focus on the aspects of treatment of HFM which lend themselves to the use of virtual surgical planning. For a more comprehensive account of the classification and treatment options of HFM, readers are referred to Adrian Sugar [14], Hemifacial Microsomia, diagnosis, classification and management, Chapter 4.5, Pages 331–388 in the recently published AOCMF book on Advanced Craniomaxillofacial Surgery, edited by Ehrenfeld M, Futran N, Manson P, Prein J, published by Thieme Stuttgart and New York, 2020, the on-line AO Surgery Reference [15] and publications on specific aspects of care.

In this paper, we will be demonstrating Planning using the following modalities:

Materialise Mimics© Innovation Suite and Depuys Synthes Proplan CMF©
3D Systems© Freeform+ (with haptic)
Stereolithographic (SLA) physical models produced from CT scans by rapid prototyping.

## Construction of missing parts of the zygomatic bone, temporo-mandibular joint and mandible [14], [15], [16], [17], [18], [19], [20], [21]

**Figure 4:**
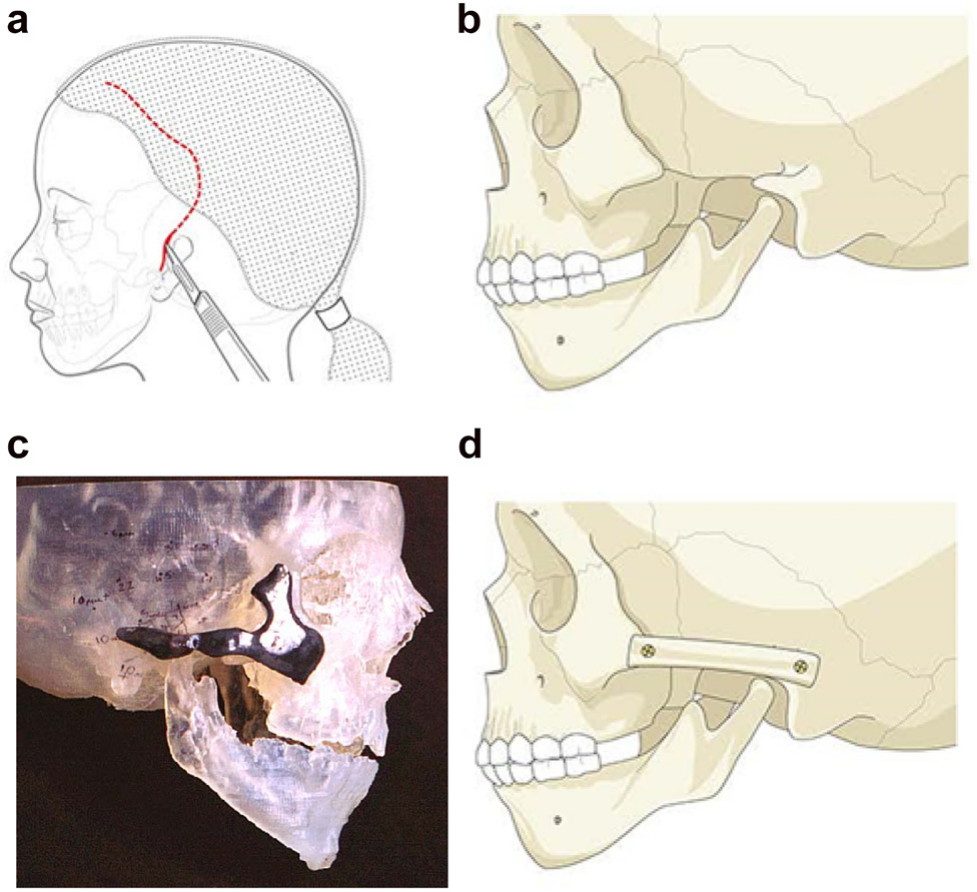
Planning for construction of missing zygomatic bone parts. (a) The placement of this incision needs to take into consideration the need for any future ear construction and avoid limiting soft-tissue cover. If a patient has microtia with limited skin and a low hairline, a temporoparietal fascial flap covering either a cartilage or porous polyethylene implant may be required. In these instances, the microtia repair should be considered first or the incisional approach modified to preserve the flap. (b) Absent zygomatic arch. (c) Guide for zygomatic arch + construction. (d) Illustration of the arch constructed.

**Figure 5:**
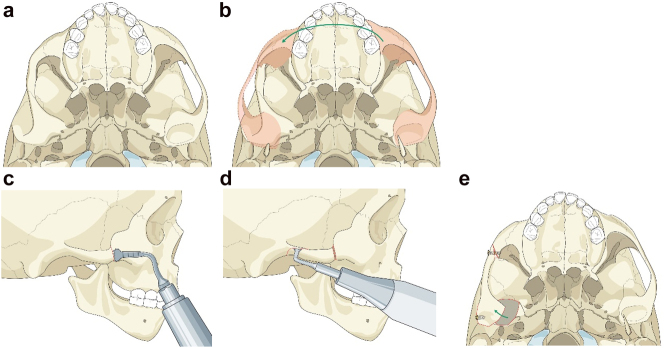
Planning for moving an aberrant glenoid fossa. (a–e) Illustrations simulating the movements required (a and b) and below the osteotomies made (c–e).

The zygomatic arch may be largely missing while the temporo-mandibular joint is present. In such cases, the missing part of the arch may be constructed with a rib graft inserted from a pre-auricular or hemi-coronal approach. This can be simulated from a CT scan in suitable 3D software to produce the right shape and curvature but with experience that is not mandatory (Figures 4 and 5).

Occasionally if the glenoid fossa is aberrant but morphologically appropriate (mostly cases of PK IIb when the fossa is usually very medially placed), it may be possible to osteotomise it and move it into a better position (laterally). The same approach as above is required. This is dependent on the amount of bone above the fossa being sufficient in order to avoid damaging the dura and temporal lobe of the brain. In the case described the minimum height of bone was 5 mm on CT scans. The use of a piezosaw to reduce the risk of damage to the dura is advisable.

When the zygomatic arch and glenoid fossa have to be constructed (Figure 6a–c) as in most PK III and many IIb cases, the same surgical approach as above is also required with the same provision that it should take into consideration the potential need in microtia cases for skin cover without scars if autogenous ear construction is to be considered in the future. It is important that the zygomatic and temporal constructions which include the glenoid fossa are a first stage (Figure 6d–i) and are not carried out at the same time as costo-chondral grafting to reconstruct the missing part of the mandible, to avoid the development of TMJ ankylosis [20]. It is also important for the same reason that soft-tissues line the construct over the glenoid fossa and whenever possible we use pedicled temporal fascia [14] if it is present. If it is absent, we use fascia lata as a two thickness layer and free graft. That can be carried out either during the first or second stage, the latter of which is usually around 6 months after the first.

**Figure 6:**
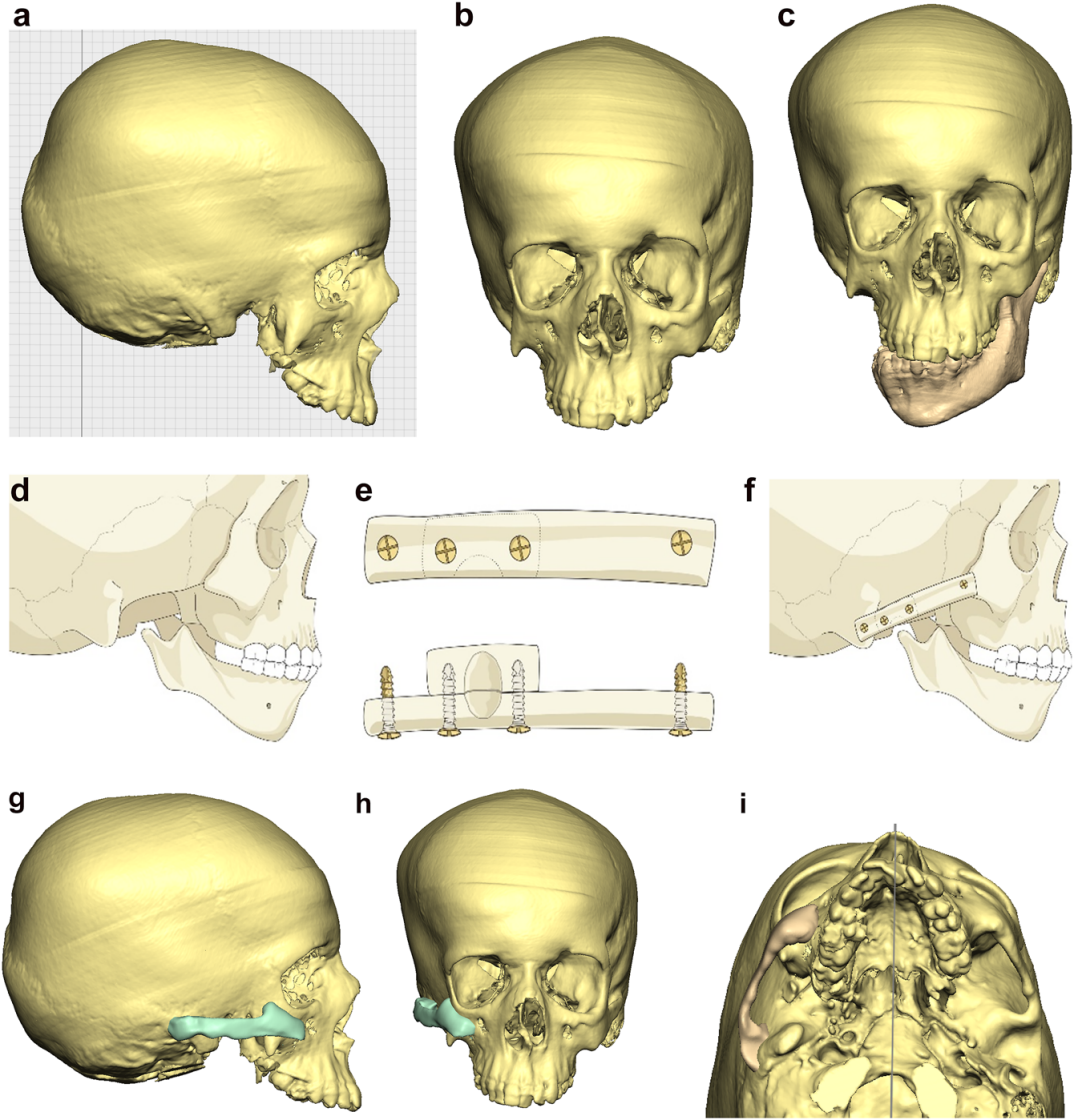
Planning for construction of missing zygomatic bone, glenoid fossa and mandible. (a–c) A severe PK III case (same case as Figure 2c) without and with the mandible. (d–f) The principle of arch/fossa construction with rib grafts. (g–i) Simulations in freeform of construction of the zygomatic arch and glenoid fossa (same case as Figure 2c, and Figure 5a–c).

When the mandible then has to be constructed (that is, when the condyle and part of the ramus, sometimes part of the body, are missing) around 6 months later, the surgical approach is usually a pre-auricular/hemi-coronal approach as above, supplemented by a submandibular approach. If the defect is small and confined to the mandibular ramus, the choice of side for rib harvest is not so important and it is best to have a reasonably straight graft. However, when the defect is large and approximates a hemi-mandible, it is helpful to choose a rib which is quite curved and from the contra-lateral side (left rib for right mandible and vice versa). Further contouring can be carried out at surgery with rib benders. Sometimes it is helpful (for example when the course and plane of the facial nerve needs to be identified) to combine these two approaches into an extended parotid or facelift approach. Although this construction applies to most cases of PK III, the same principle applies to many cases of PK IIb. The length of the rib graft is not only dependent on the length of the defect. Often it has to be extended in order to reach bone for fixation where there is sufficient space without damaging buried unerupted teeth (see Figure 6o and p).

**Figure 6:**
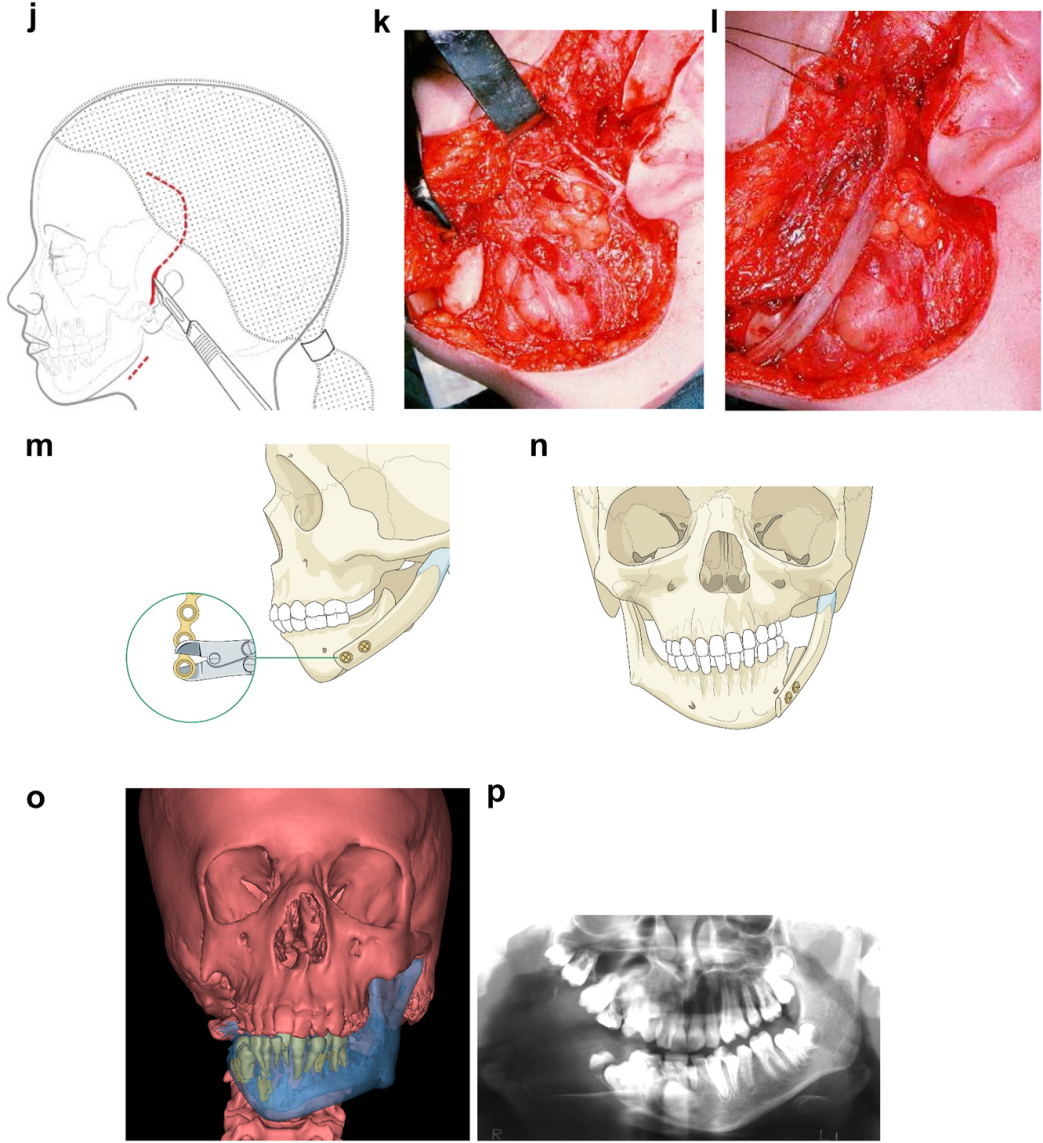
Planning for construction of missing zygomatic bone, glenoid fossa and mandible. (j) The surgical approach. (k) The facial nerve dissected. (l) A long costo-chondral graft inserted (Figure 6k and j are a different case from the rest of Figure 6). (m and n) Illustrations of the grafts inserted and fixed and a defect between the mandible and rib graft filled with further rib graft. (o and p) Transparency 3D CT view in Mimics and OPT of same case as Figure 2c showing the buried teeth and roots in the mandible indicating that the costo-chondral graft construct has to be extended anteriorly over the chin to permit safe fixation with two screws.

**Figure 6:**
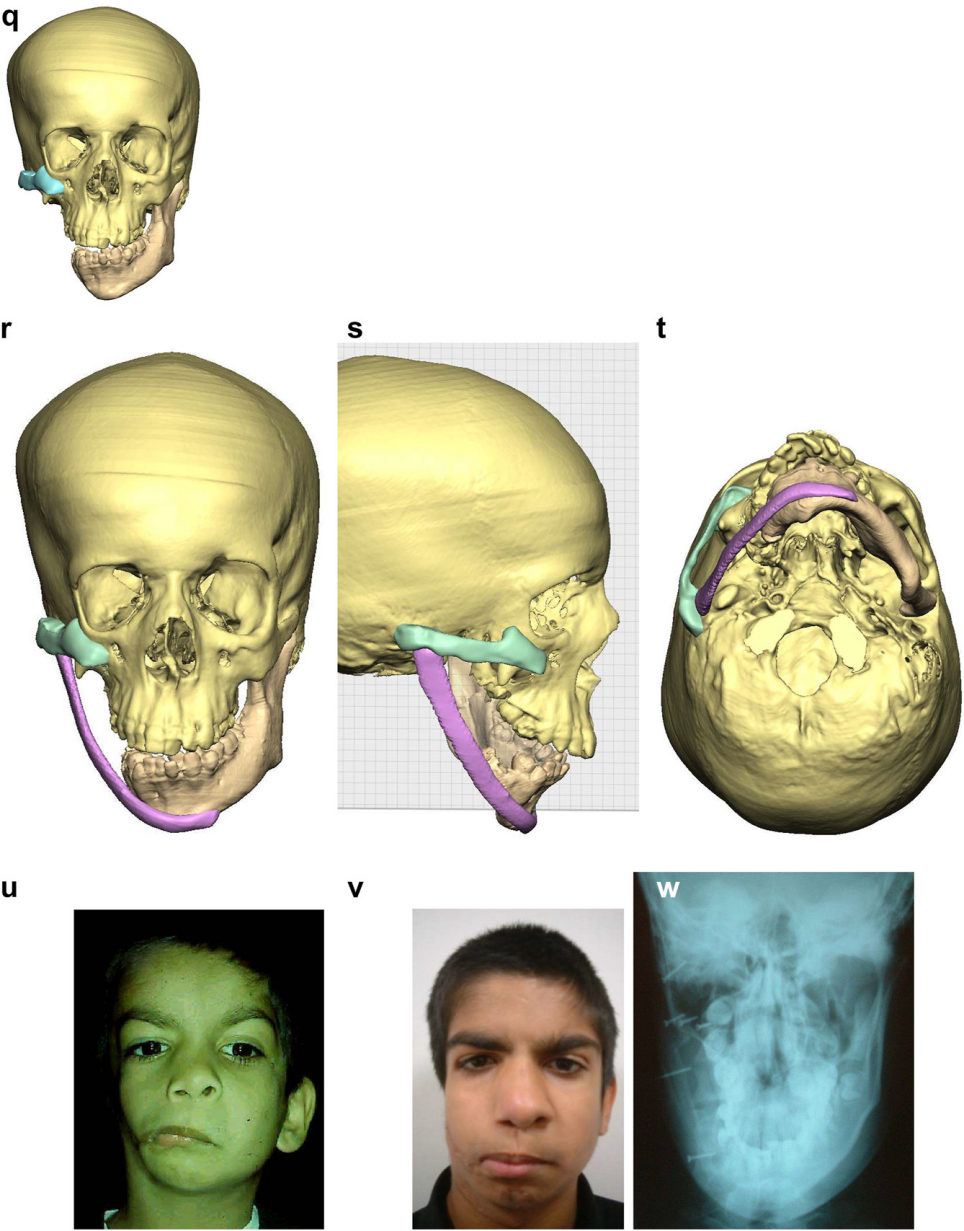
Planning for construction of missing zygomatic bone, glenoid fossa and mandible. (q) Simulation in freeform of the mandible having been swung to the left hinged on the normal left TMJ. (r–t) Simulation in freeform of contoured right costo-chondral graft to construct the right mandible (harvested from the left 6th or 7th rib which assists the correct contour). These constructs may be 3D printed and sterilised to act as a guide for contouring of the rib grafts during surgery or 3D sterilisable guides are made. It should be noted that there are potential areas of undesirably contact between the mandibular rib graft and the stump of the residual zygomatic bone and the mastoid bone and these need to be addressed at surgery. Also in these long reconstructions of the mandible, there is commonly a v-shaped defect at the point where the graft approximates to the mandible. It is good to pack that defect with further pieces of rib as shown in Figure 6n and Figure 7b. (u) Same case as Figure 2c and most of Figure 6 PK III with incomplete Tessier no. 7 facial cleft, Figure 6. (v) Clinical outcome Figure 6. (w) Radiographic follow-up.

**Figure 7:**
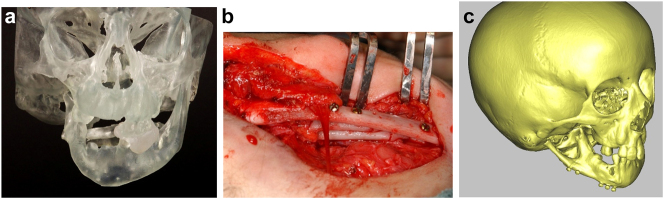
Construction of mandible with rib grafts. (a–c) A PK IIb case with long costo-chondral graft and further rib grafts to fill the defect with CT outcome.

In severe type PK III deformities in which the patient is missing not only the ramus but a portion of the body, a vascularized fibula is considered by some surgeons to be the procedure of choice [21, 22], due not only to the length of the defect, but also the need to create a gonial angle. For planning, a 3D CT scan is required not only of the face but of the donor fibula as well. After repositioning or centring the mandibular midline a two piece construct is envisioned. A cutting/drill guide is fashioned as is a custom 3D printed mandibular reconstruction plate. During surgery the flap is dissected out while still attached to its vascular pedicle, and the osteotomies are completed and the plate is attached. Once the recipient site is prepared, which includes a drill guide for the native mandible, the flap is transferred and the microvascular transfer completed (Figure 8).

**Figure 8:**
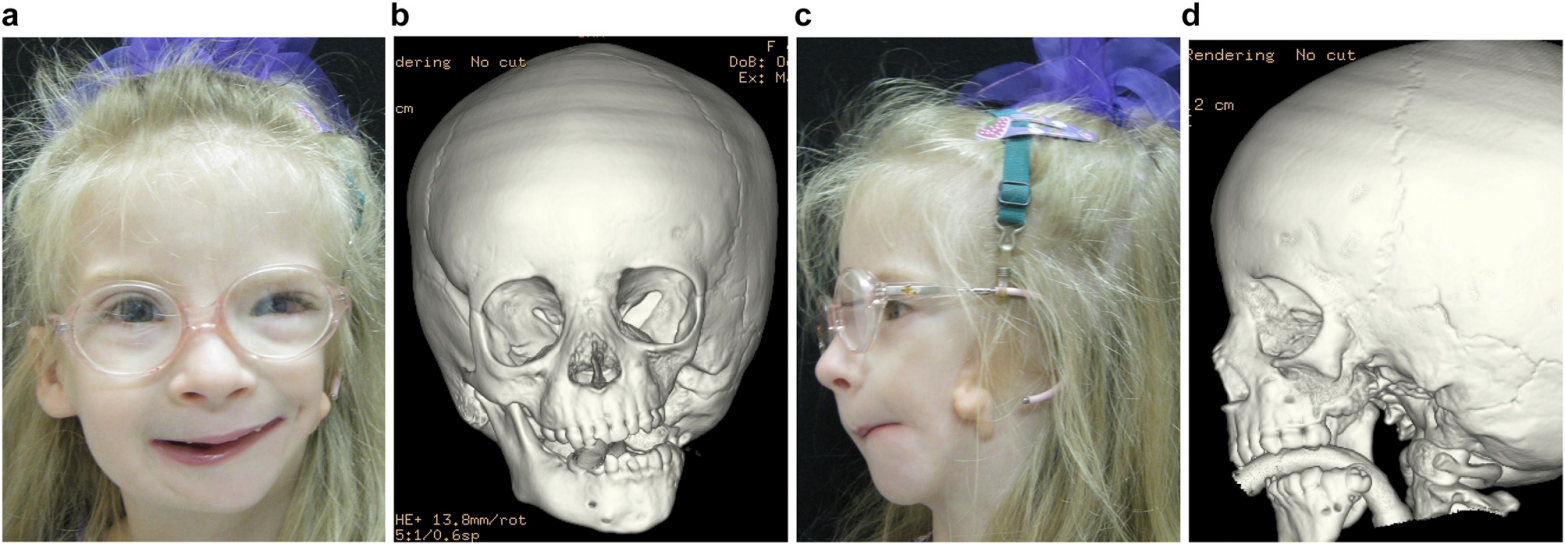
Construction of mandible with fibula free microvascular flap (a–d) 6 yr old girl with PK III HFM.

**Figure 8:**
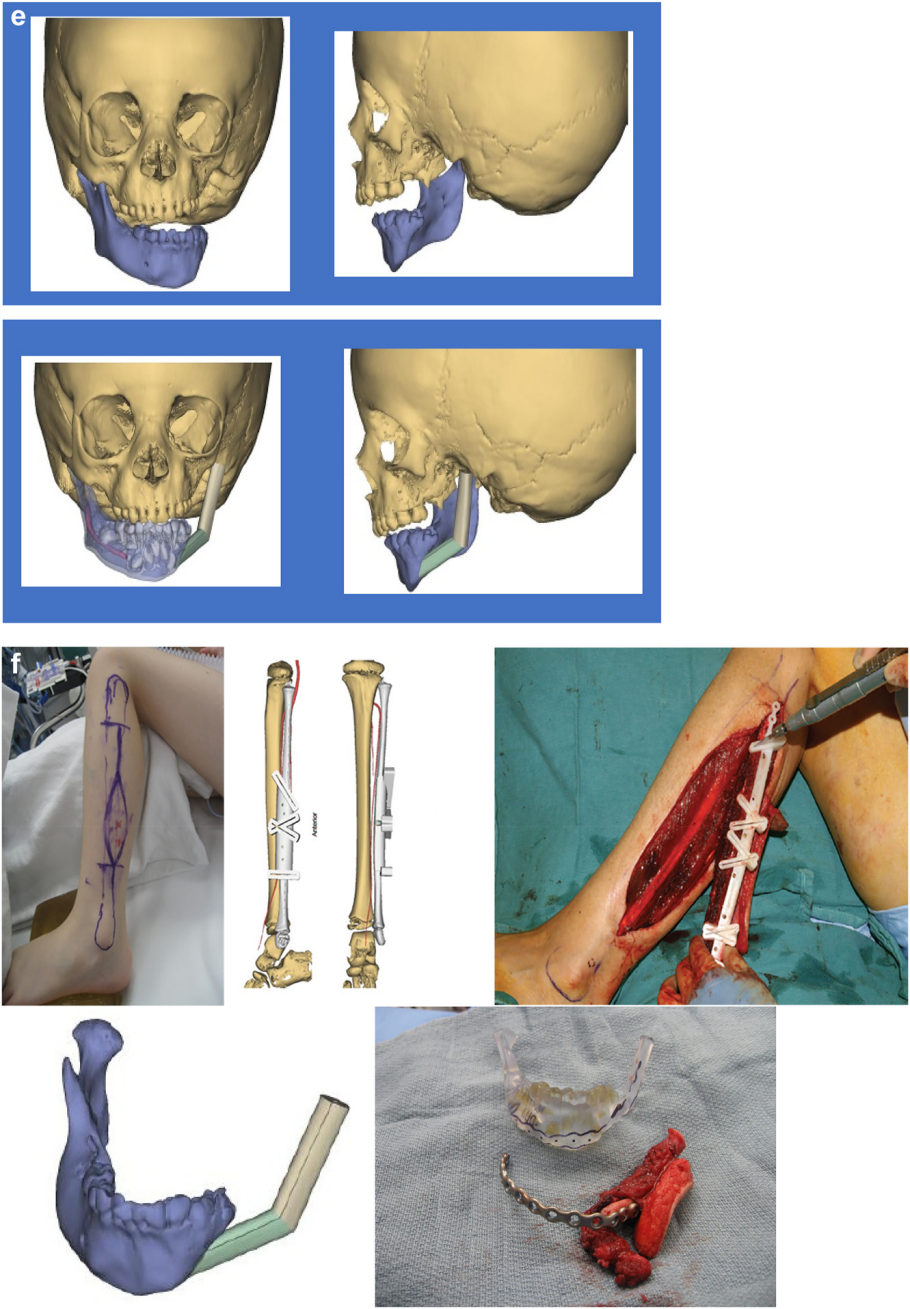
Construction of mandible with fibula free microvascular flap. (e) Virtual plan for fibula free flap. Harvest of fibula free flap using 3D printed guide and saw. (f) The fibula harvested with 3D printed guide, virtual 3D construction, 3D printed model of that plan, and bent reconstruction plate attached to fibula before flap vessel anastomosis.

**Figure 8:**
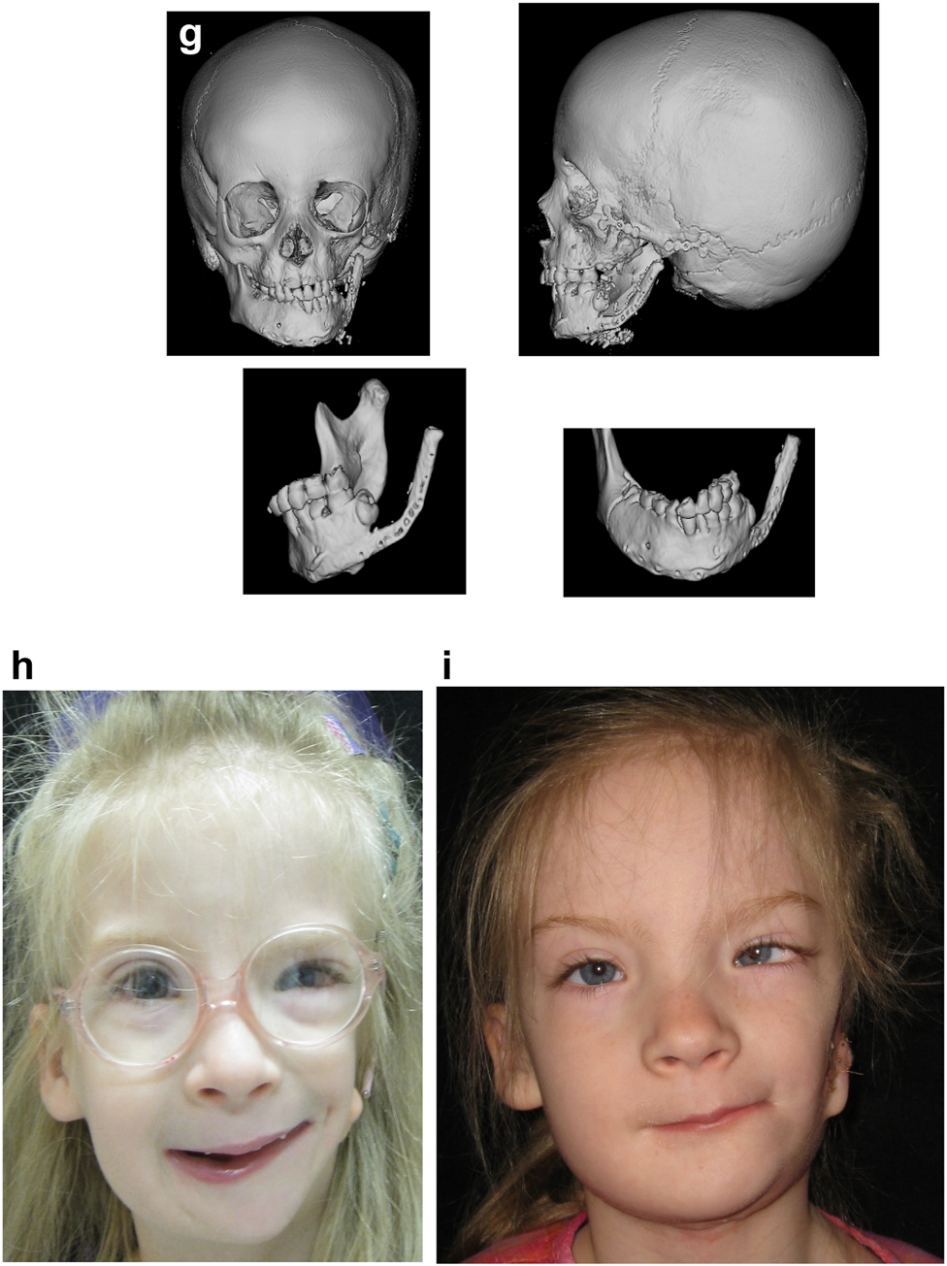
Construction of mandible with fibula free microvascular flap (g) 3D CT Outcome of the construction. (h) Pre-op. (i) Post-op.

## Virtual planning for distraction osteogenesis for ramus lengthening [14, 15, 17, 18]

Having now reconstructed the missing parts of the mandible and TM Joint, if all goes well these structure will be complete. Sometimes the free bone grafting may need to be supplemented with further grafts. The main aim is to convert a PK III case (by definition with missing mandibular condyle with or without missing zygomatic structures) or a IIb into a PK IIa with a deformed but intact mandible (in the OMENS Classification converting an OMENS M3 and an M2B into an M2A). The mandible and these structures may then grow with the patient normally and some time needs to be allowed to assess this. Sometimes we will use the costochondral graft to rotate the mandible towards the unaffected side to minimise the asymmetry. We do not however use the graft to lengthen the mandible vertically on the affected side mainly because it is rarely possible to gain sufficient length. If vertical growth on the affected side proves to be inadequate, we try to achieve that vertical increase in length with distraction osteogenesis.

The same principle applies to patients with HFM classified originally as PK IIa cases whose mandible is small and misshapen but essentially intact. This is demonstrated in the next plan and clinical case.

The DICOM CT images are imported into the software. On the 3D image of the affected side ramus, in the virtual planning a distractor is chosen and positioned in the preferred position to produce vertical lengthening in the desired vector of distraction going through the condylar process. The osteotomy is chosen and completed. The distractor is then virtually activated to the amount to achieve the desired correction. If the vector and/or correction are not appropriate, the distractor is repositioned and the process restarted. In each case, the plan can be saved. Once the plan is agreed, a virtual guide can be produced incorporating a shape which will locate on the ramus, holes for the distractor screws and to stabilise the guide and a groove for the osteotomy cut.

## Virtual planning for distraction osteogenesis for correction of lateral facial contour [14, 15]

**Figure 9:**
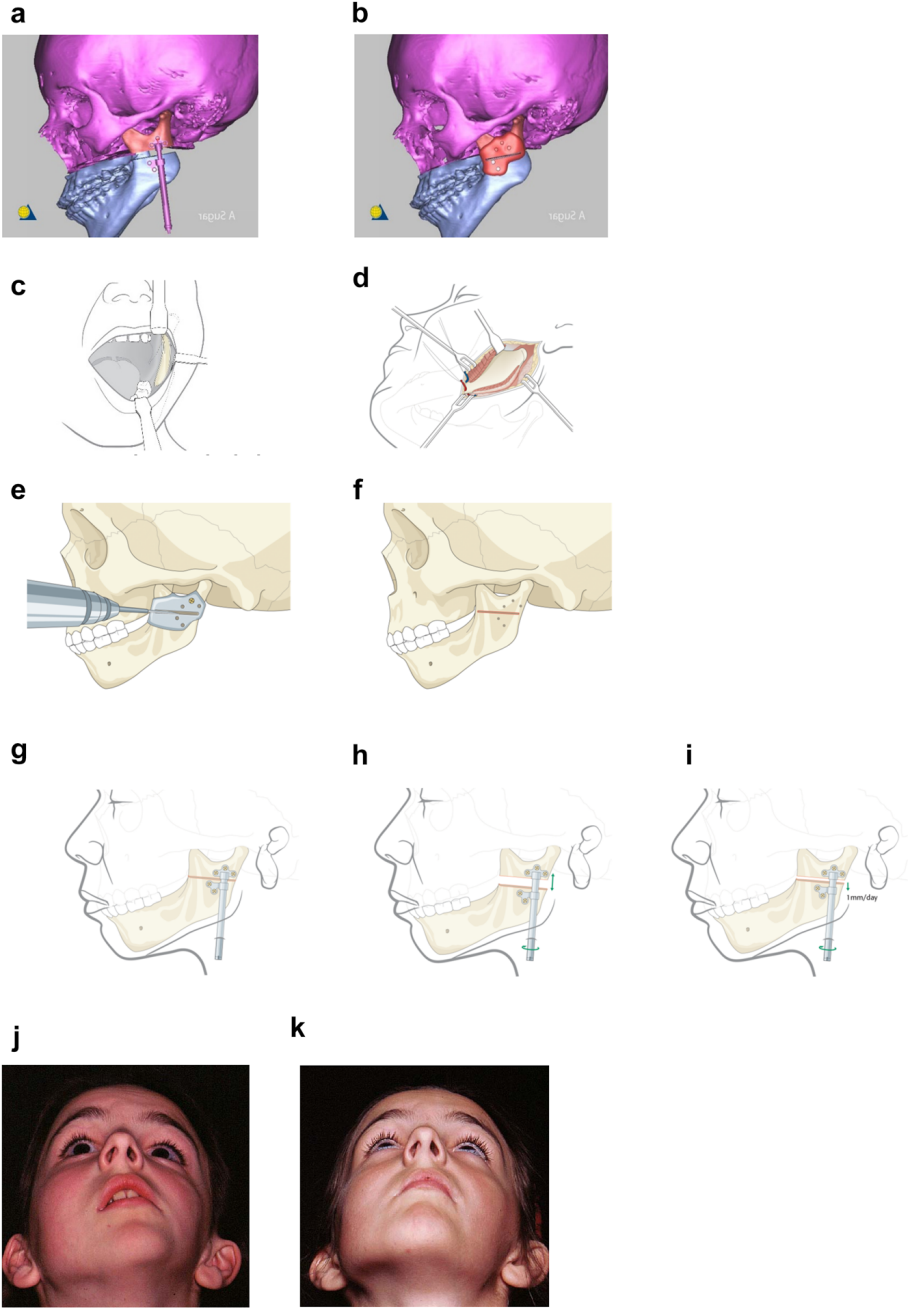
Planning for Distraction Osteogenesis (DO) to vertically lengthen the mandibular ramus. (a) A virtual distractor in position. (b) A virtual guide for this distraction which is 3D printed in metal before sterilisation. (c) An oral approach which is supplemented by trans-buccal instrumentation. (d) An external skin approach. (e) The guide is introduced and stabilised with a single screw. The holes for the distractor screws are drilled trans-buccally. The groove for the osteotomy is made with a reciprocating saw (for an external approach, an oscillating saw is used). (f) The guide has now been removed and the osteotomy cut is completed. (g) The distractor is inserted and fixed with screws. (h) The distractor is test-activated and then returned to its original position. (i) Distraction proceeds after a few days latency at 1 mm per day. (j) Pre-op. (k) After left vertical ramus distraction (same patient as Figure 1a and b).

**Figure 10:**
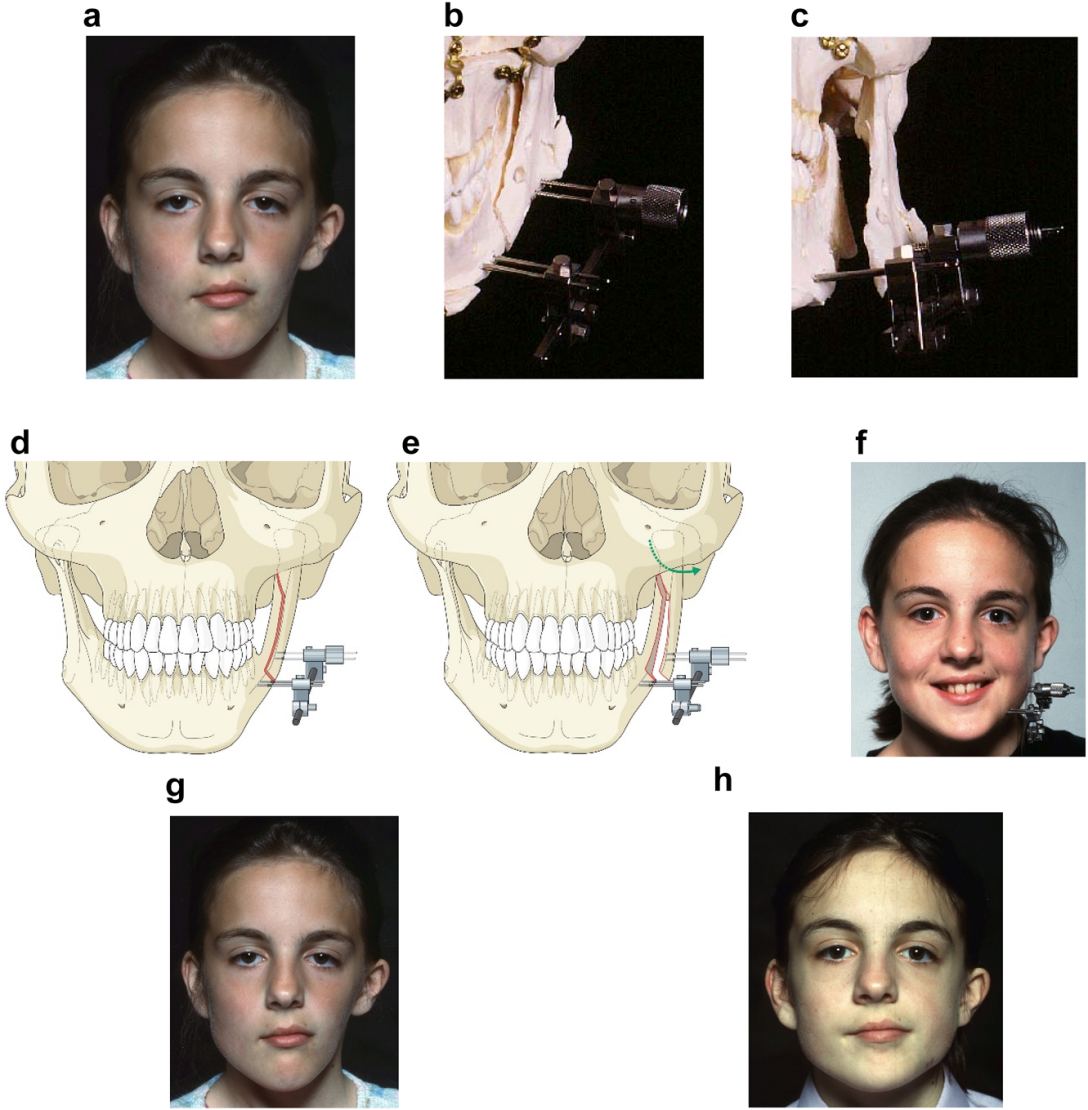
Planning for DO to broaden the mandibular ramus (same patient as Figure 9). (a) 1 year after left vertical DO. (b and c) Pins and distractor *in situ* on model with sagittal split osteotomy of left mandibular ramus with physical simulation of lateral DO. (d and e) Diagrammatic simulation of lateral distraction. (f) During lateral DO. (g) Post-vertical DO with lateral contour deficiency. (h) After both vertical and lateral DO.

Occasionally in HFM cases, the one aspect that needs to be addressed is lack of lateral contour of the face, even after vertical ramus distraction (DO). The above patient (Figure 9), who was referred at 12 years of age, underwent orthodontics followed by vertical ramus distraction, and was an example of this. One year after vertical distraction she returned very upset by the lack of contour of the face on the affected left side as demonstrated below (Figure 10a).

We considered various options for correction including onlay augmentation with bone, cartilage, dermis fat, fat injections or an alloplast. We decided instead to perform lateral distraction but since that had not been described previously, we tested it first by planning on a physical model. The aim was to carry out a sagittal split osteotomy on the affected (previously distracted) side, and insert a distractor with mono-cortical pins in the anterior part of the condylar fragment, and bi-cortical pins in the body of the mandible anterior to the osteotomy. These were connected by a distractor which drew the posterior pins laterally until satisfactory contour was achieved. The screws which held the posterior pins to the distractor were only loosened immediately before distraction with the patient holding her teeth together and were then tightened after the distraction. The daily rate of distraction was 1 mm and no inter-maxillary fixation was required.

## Virtual planning for distraction osteogenesis for ramus lengthening when the lateral ramus contour of the mandible is unfavourable

The principle of using distraction osteogenesis for vertical lengthening of the mandibular ramus is the same whether the patient has had mandibular construction (PK III and IIb cases), or was originally IIa as shown above (Figures 9 and 10). The principle is the same, but the practise may be a little different.

**Figure 11:**
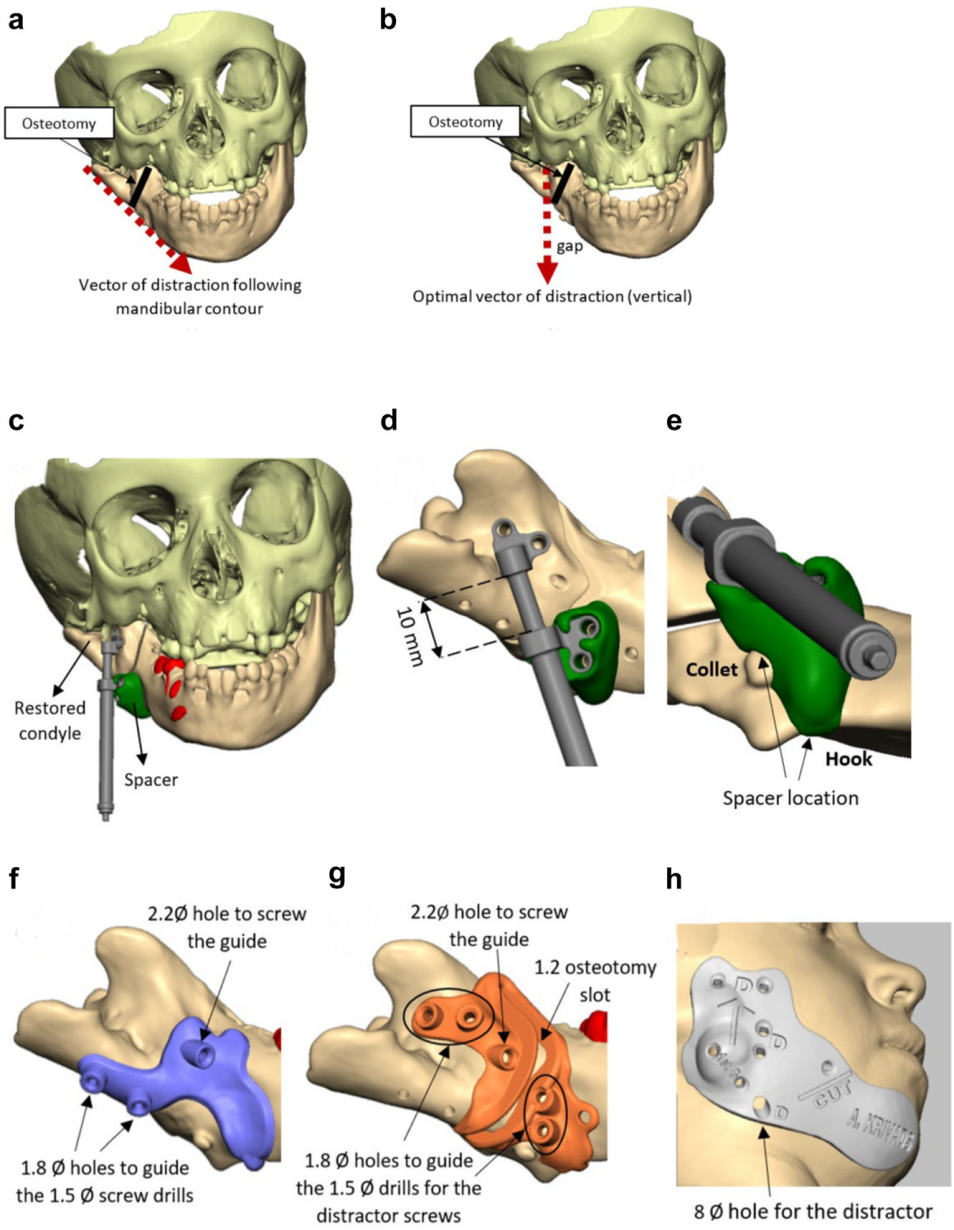
Planning for DO to vertically lengthen the mandibular ramus when the ramus is deficient laterally. (a and b) The vector problem in this reconstructed case. (c–e) The planned solution correcting the distraction vector to vertical with a spacer. (f–h) The virtual guides.

**Figure 11:**
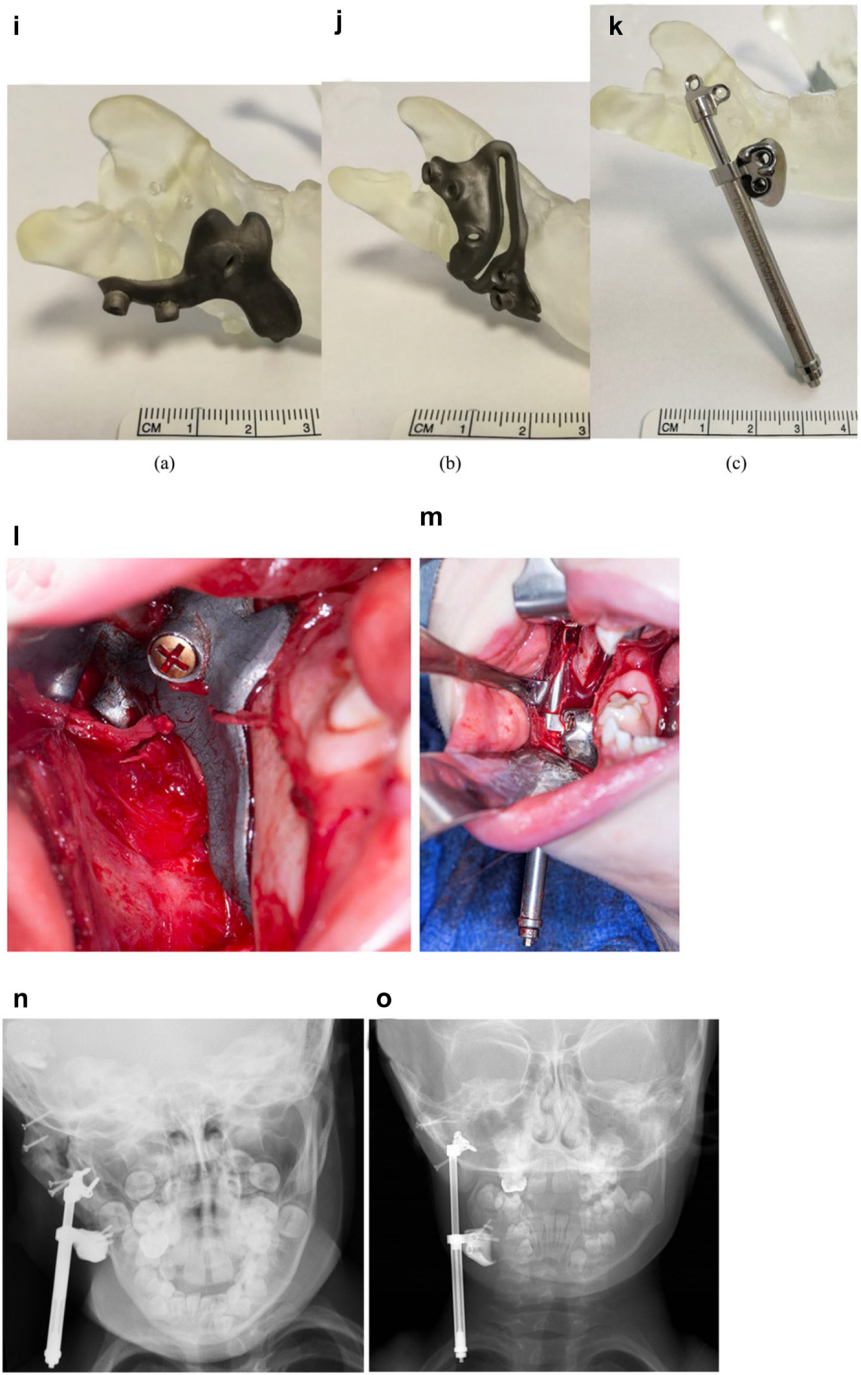
Planning for DO to vertically lengthen the mandibular ramus when the ramus is deficient laterally. (i–k) The 3D printed guides, spacer and distractor. (l and m) During surgery. (n and o) PA radiographs of the start and end points of distraction.

In previously constructed cases, we carry out the distraction itself within the original part of the mandible (see Figure 20g), not in the consolidated rib graft, as the original part of the mandible to which the previous rib was attached is likely to be more robust. Also it is usually necessary in such cases to remove some metalwork (usually screws) in the planning process and at surgery. The morphology of the lateral mandible on the affected side may be unfavourable preventing a vertical distraction. We have developed a method to address that [23] which we describe below (Figure 11) in a PK IIb case which had been constructed by us previously with a costo-chondral graft.

**Figure 12:**
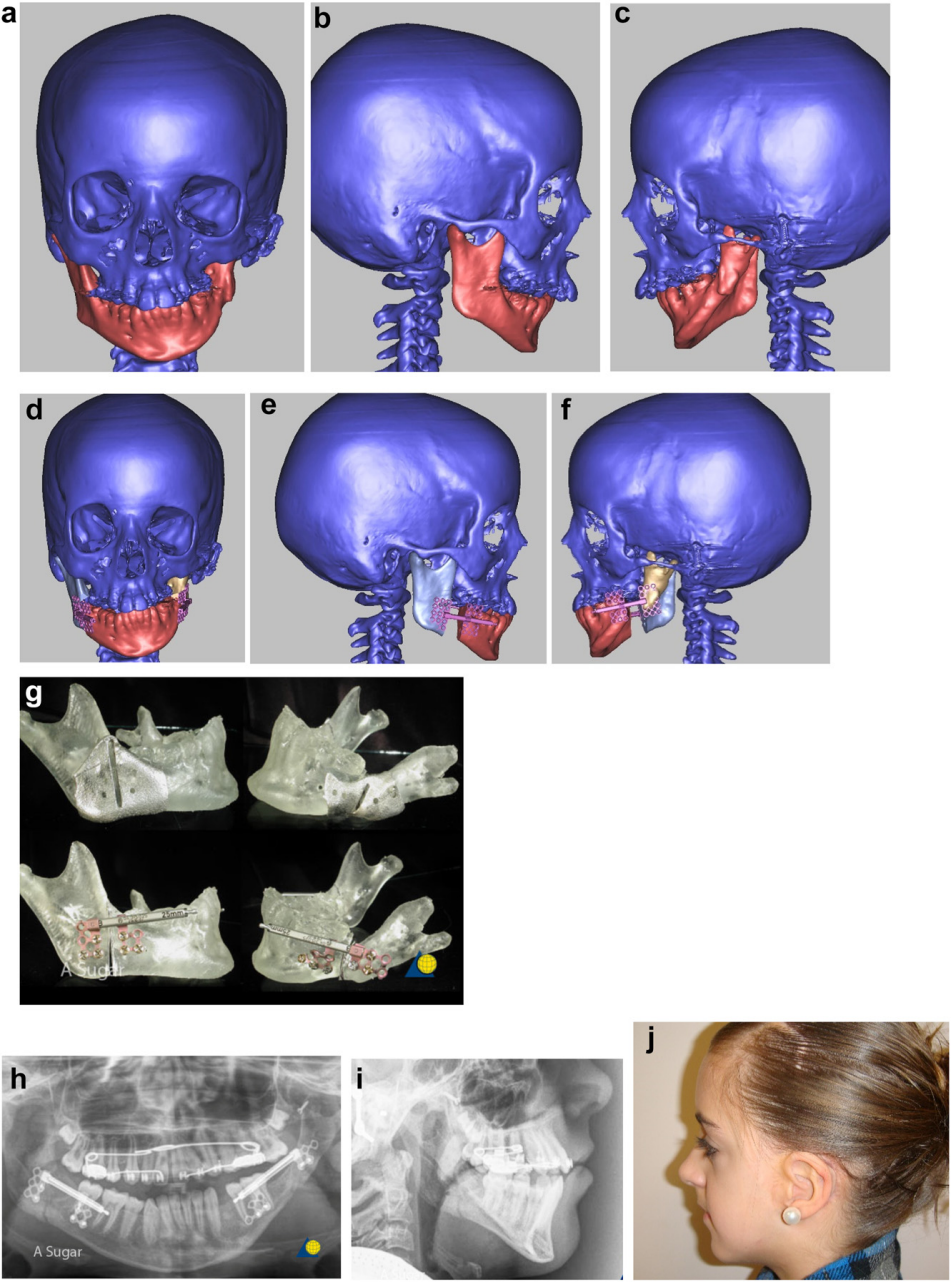
Planning for DO to advance the mandible horizontally. (a–c), (d–f) Virtual distractors in position and activated. From these, virtual guides were produced and 3D printed in metal (Figure 13g). (g) Actual guides and distractors on physical model. (h) End-point of distraction. (i and j) Outcome of bilateral mandibular body distraction – note left bone anchored ear prosthesis *in situ*.

**Figure 13:**
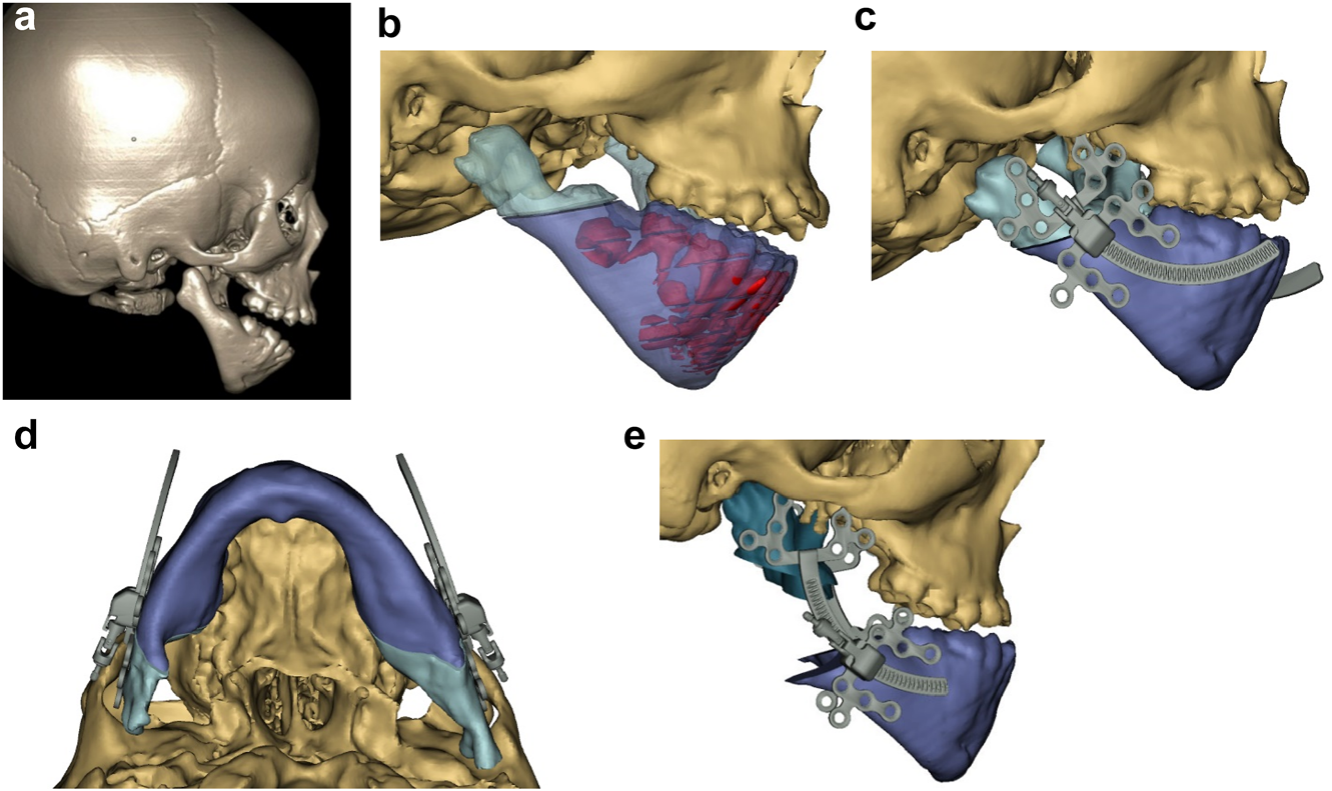
Planning for Curvilinear DO to lengthen the ramus vertically and to advance the mandible (a–e).

## Virtual planning for distraction osteogenesis for mandibular body advancement

Growth after early interventions may be unfavourable and in this case gave concern to the young lady who was significantly retrognathic with some remaining mandibular asymmetry (see Mimics CT images, Figure 12). She requested further correction and it was decided to advance the mandible bilaterally with intra-oral distraction. The virtual plan is below.

## Virtual planning for curvilinear distraction of mandible

This technique is particularly useful for cases which require vertical lengthening of the ramus and advancement of the mandible, especially when bilateral and often needing closure of an anterior open bite (Figure 13). The planning process is done in reverse, that is the new position of the mandible is chosen after osteotomies, the arc of the curvilinear distractors is determined and the virtual simulation carried out with those as seen below. The process of insertion, osteotomy and activation are challenging.

**Figure 14:**
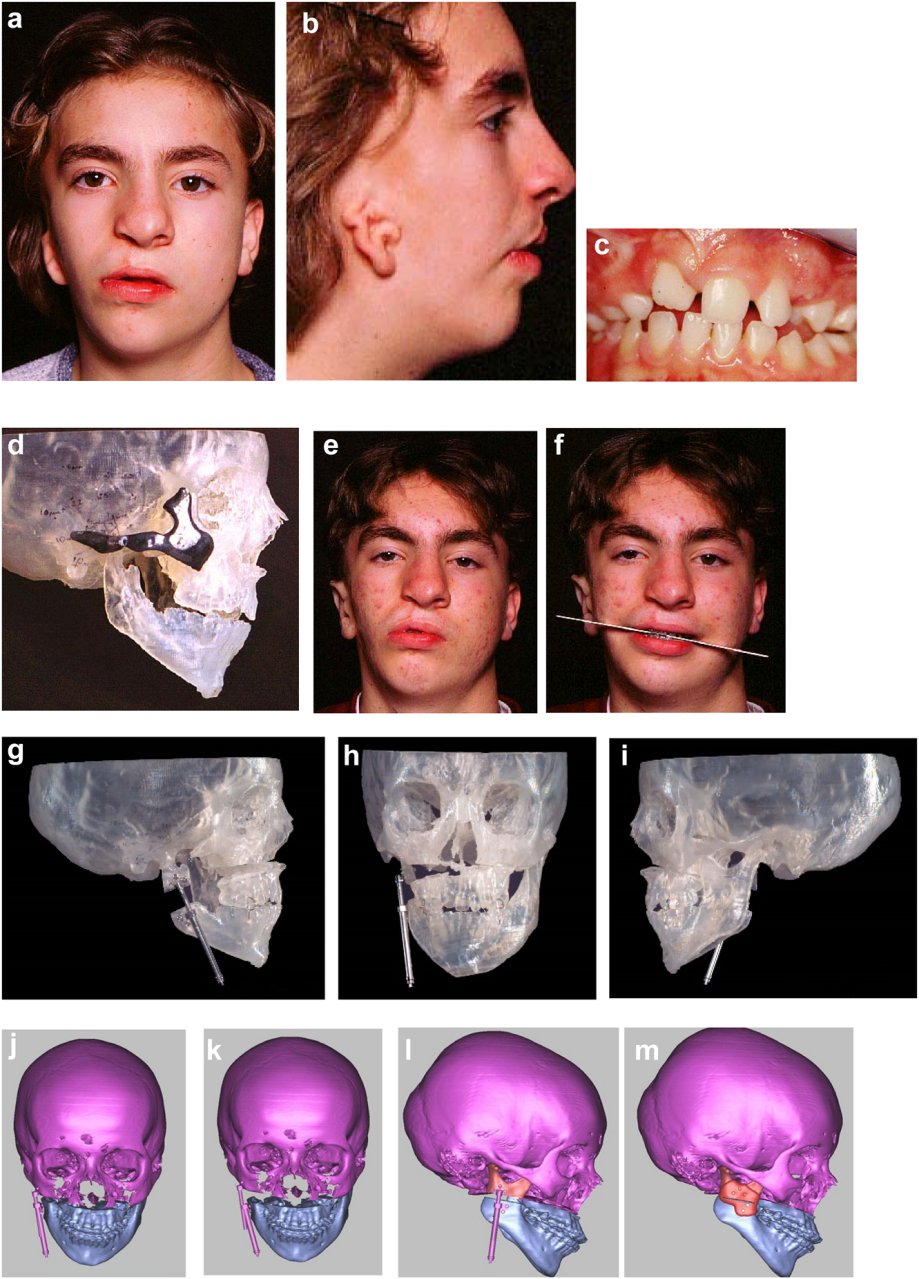
Planning for bimaxillary DO. (a–c) 12 year old male with PK IIa HFM and a repaired right sided UCLP. (d–f) Guide for zygomatic augmentation with rib graft and measurements for bone anchored ear prosthesis with outcome of both. (g–i) Plan on SLA model from CT scan. The maxillary attachment at the nasal septum is retained and the left maxilla rotates upwards during distraction for which bone needs to be removed. (j–m) Virtual bimaxillary distraction plan in another patient.

**Figure 14:**
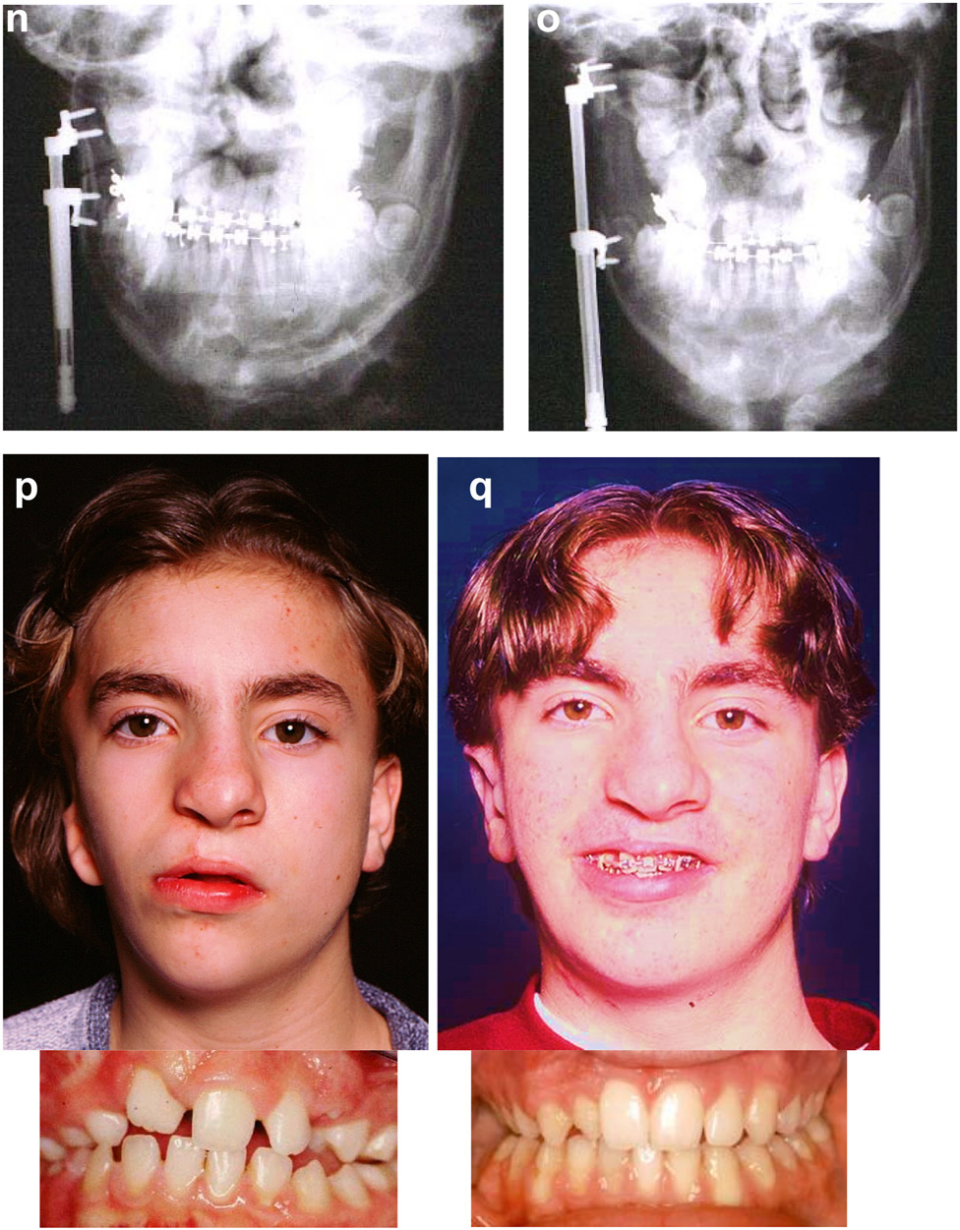
Planning for bimaxillary DO. (n and o) Radiographic outcome in case in Figure 12a and b – pre-distraction orthodontics has been carried out. (p and q) Before and after orthodontics and bimaxillary distraction.

## Virtual planning for bimaxillary distraction osteogenesis

When correction of asymmetry in HFM is required in the age group 12 yrs to early teens, a facial rotation is usually needed including the maxilla to avoid a potentially permanent lateral open bite on the affected side, since the maxillary teeth may not erupt into the space created by the vertical mandibular lengthening. In these cases, a simultaneous bimaxillary distraction may be considered as described by Ortiz Monasterio [24] and also by Padwa [25]. This may be considered end stage and in our experience has avoided later rotational osteotomies. We have chosen to do this only in cases with a Class I occlusion or in which orthodontics can create a reasonable occlusion.

As with lateral distraction described above, for our first case we chose to plan this using physical SLA models (from the patient’s CT scan) which we describe below but this can also be done virtually as also described (Figure 14). During distraction and consolidation, the teeth are held together with inter-maxillary elastics using the orthodontic devices, the distraction in the mandible drawing down the maxilla with it.

## Virtual planning for bone anchored ear prostheses

Reconstruction of microtic ears in HFM are generally carried out in experienced specialist units by either bone anchored ear prostheses [26], [27], [28], [29] (BAEP) or autogenous ear construction [30], [31], [32] (AEC). The age for BAEP will largely depend on the cooperation of the child but can be as early as 3 yrs. AEC is generally delayed until 10 yrs or older. The use of porous polyethylene auricular prostheses covered by temporo-parietal fascia flaps and full thickness skin grafts is gaining popularity as a third method of construction but is beyond the scope of this manuscript. We will describe virtual planning for BAEP and AEC (Figures 15 and 16).

**Figure 15:**
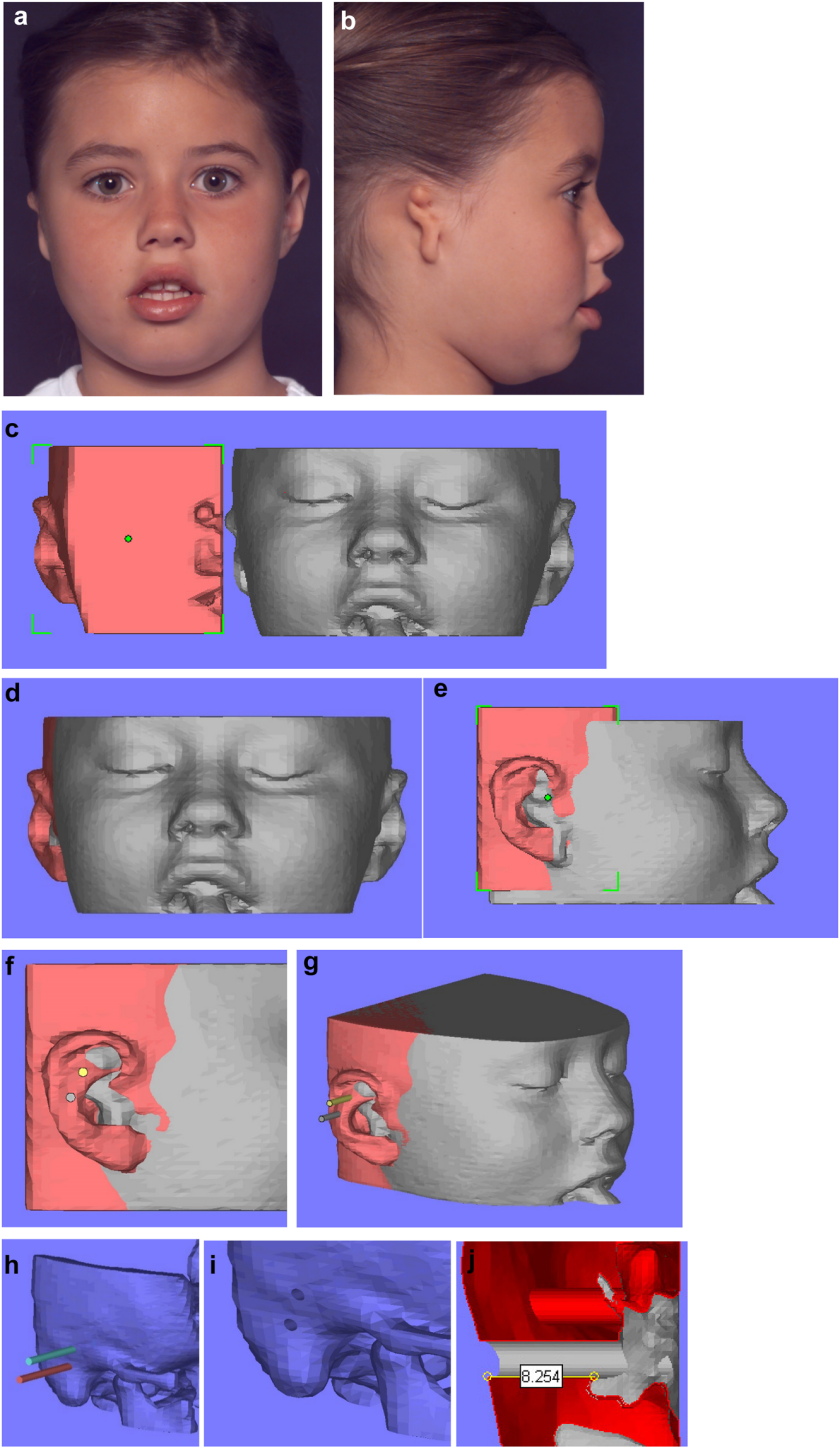
Planning for a child with microtia for a bone anchored ear prosthesis. (a and b) Right microtic ear. (c) CT scan of same patient imported into Mimics. Mirror image created (bone and soft-tissue) of left normal ear and surrounding structures and transferred to microtic side. (d and e) Mirror imaged ear moved until it matches the normal side in all dimensions. (f and g) Implants or cylinders are inserted in the appropriate sites. (h–j) The soft-tissues are then removed from the virtual image, the cylinders removed from the bone, and sections examined of the holes to ensure they have sufficient depth of good quality bone for implant insertion.

**Figure 15:**
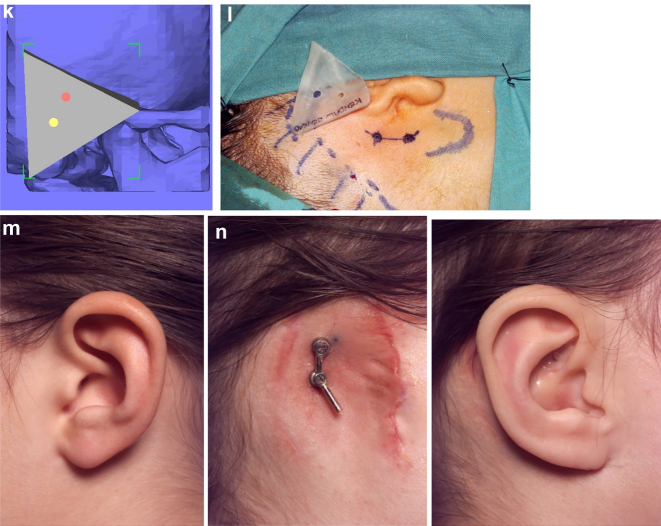
Planning for a child with microtia for a bone anchored ear prosthesis. (k and l) A virtual guide is then made (bone fitting or soft-tissue fitting) and then 3D printed as shown with the positions for the implants. (m and n) The clinical outcome, some ear cases are more challenging.

**Figure 16:**
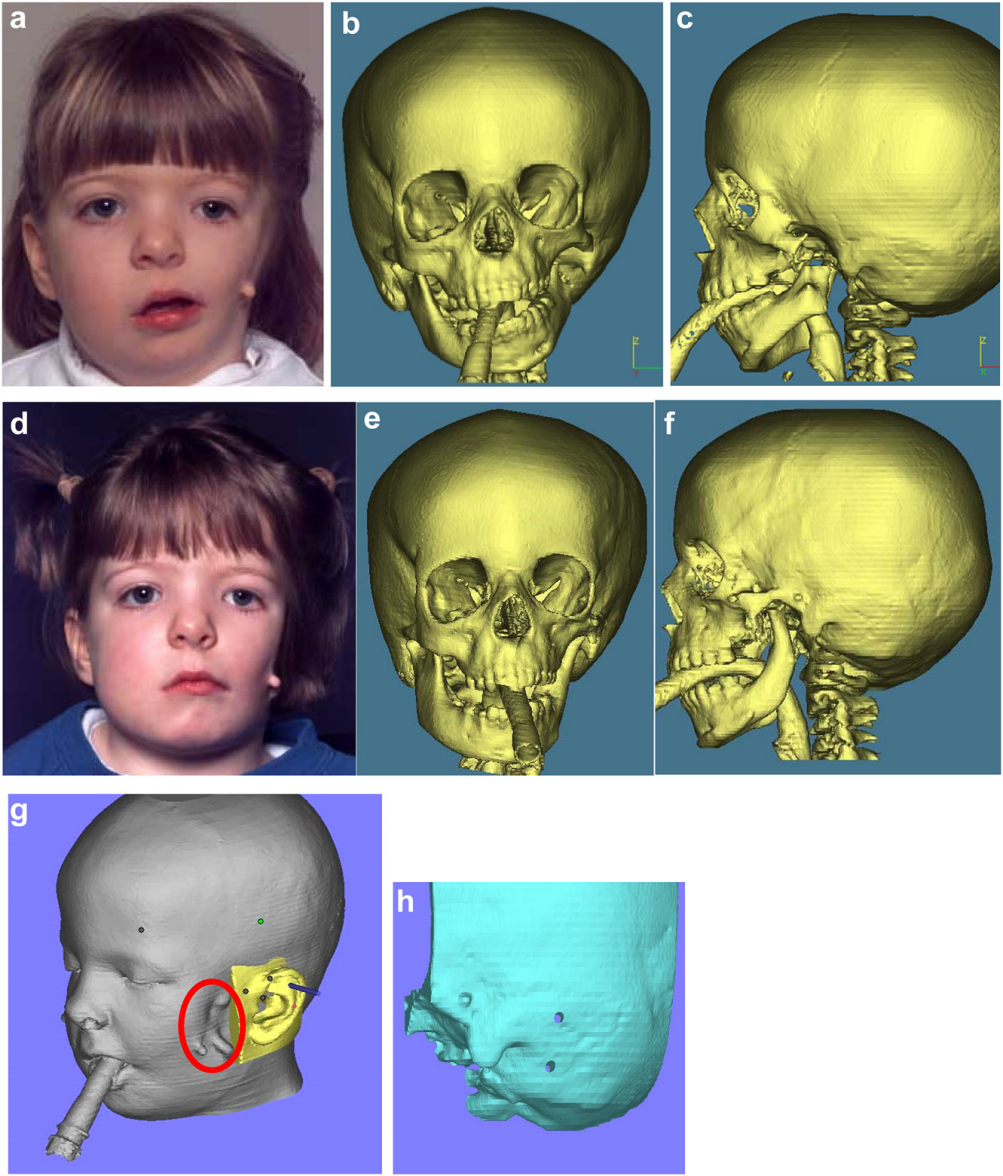
Construction of a PK III HFM child with a costo-chondral graft and then DO ramus lengthening followed by planning of a difficult bone anchored ear prosthesis with significant soft-tissue deficiency. (a–c) This young lady (Figure 16) presented aged 4 years with PK III HFM, severe scoliosis, an epi-bulbar dermoid cyst and severe microtia. She first underwent spinal fusion at 4 yrs of age, six months later construction of the left zygomatic arch and glenoid fossa, 6 months after a costo-chondral graft to construct the mandibular ramus and condyle and subsequently vertical distraction to lengthen the ramus. (d–f) Following the mandibular reconstruction and DO, g Virtual ear planning with soft-tissue Figure 17. (h) Note ideal sites for the ear/implants well posterior to the mastoid deficiency highlighted in red ellipse.

**Figure 16:**
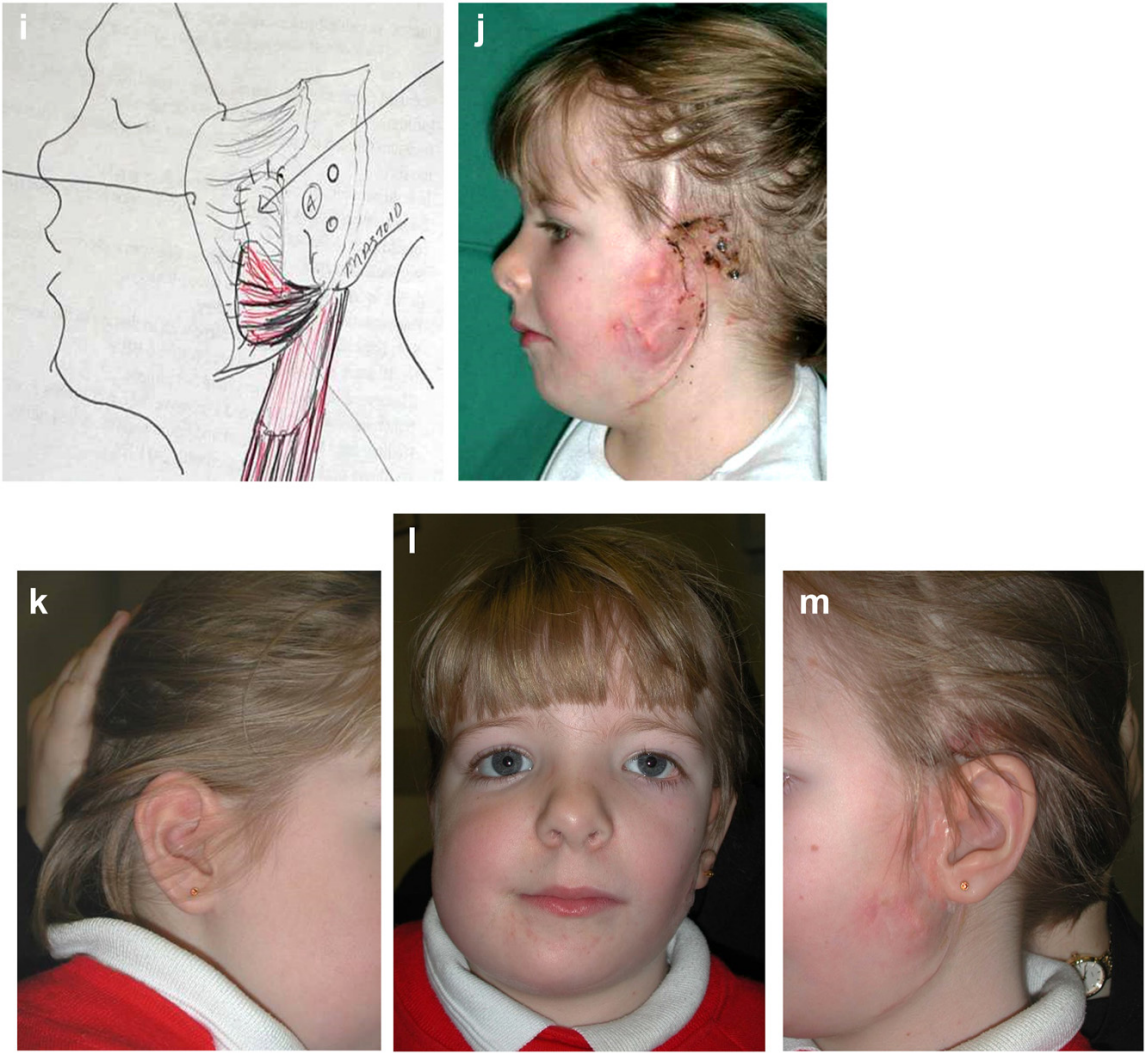
Construction of a PK III HFM child with a costo-chondral graft and then DO ramus lengthening followed by planning of a difficult bone anchored ear prosthesis with significant soft-tissue deficiency. (i and j) Note augmentation of the sub-cutaneous tissues anterior to the ear site by rotating upwards of a partial thickness flap of SCM muscle and rotating anteriorly a flap of subcutaneous tissue from around the implant sites. (k–m) The augmentation of the pre-auricular soft-tissues on the left enabled the prosthetist to construct a bone anchored ear prosthesis whose anterior margin was well disguised.

**Figure 17:**
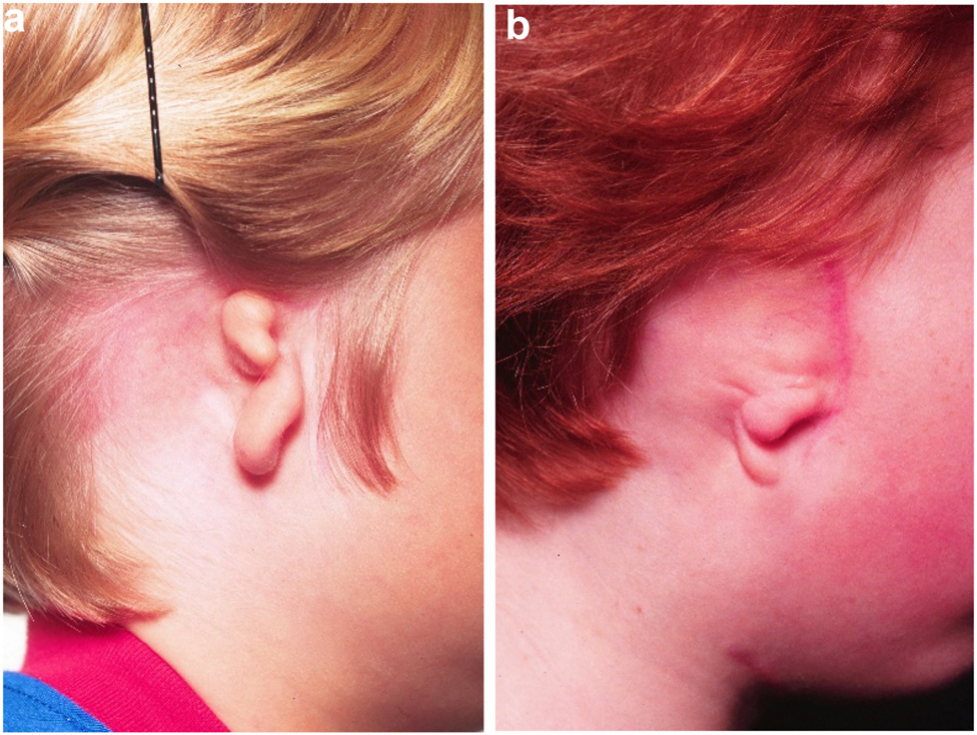
Microtia examples. (a and b) Typical microtic ears.

## Virtual planning for autogenous ear construction

A template is traced of the unaffected ear using clear plastic film (Figure 18a). The template is reversed and positioned on the affected side in the most satisfactory position based on the opposite side ear position, the hair line, and the microtic remnants which may be used to construct the ear lobe (Figure 18b). Costal cartilage is harvested from ribs 6–10 (Figure 18c) and the cartilage framework is carved to include the base and helical rim (Figure 18d). The recipient site is prepared by undermining the skin with or without transposition of the lobe (Figure 18e and f). The cartilage construct is placed subcutaneously together with a suction drain.

**Figure 18:**
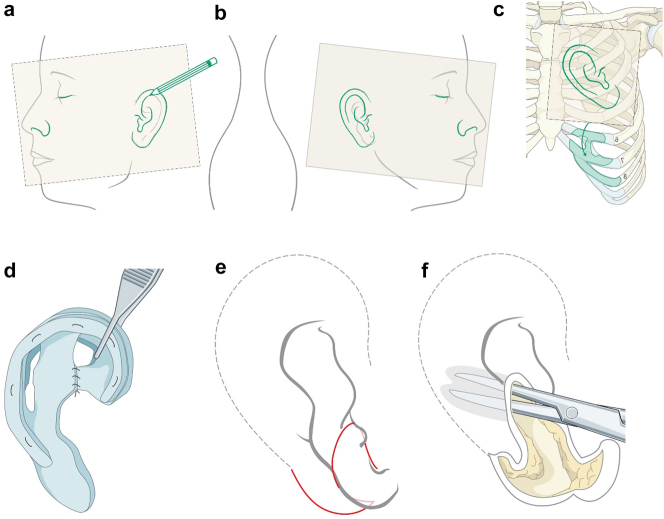
Planning for autogenous ear construction (a–f).

After stage one healing is complete, and if the lobule has been moved in stage one, the ear is elevated and additional cartilage is used to project the ear (Figure 18g and h). This creates a soft-tissue deficiency which is closed with a turnover mastoid flap or temporal parietal flap and a skin graft. Additional soft tissue rearrangements are performed in the lobule area if needed (Outcome in Figure 18i).

**Figure 18:**
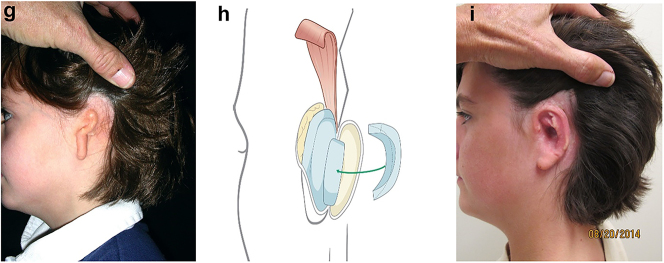
Planning for autogenous ear construction (g–i).

## Virtual planning for rotational bimaxillary osteotomies

Some HFM patients at end stage (late teens and onwards) will require rotational bimaxillary osteotomies including some of those who have had earlier surgical interventions [33]. Commonly these will be levelling Le Fort I maxillary osteotomies with rotation, inverted-L mandibular ramus osteotomy with advancement, lengthening and bone grafting on the affected side, and sagittal split ramus osteotomy on the unaffected side, these also correcting the jaw base relationship and occlusion. Often they will also require a rotational genioplasty and further bone grafting. Of course there are exceptions which can be managed with more limited procedures or none and bilateral cases which will be managed according to their particular dysplasia.

The challenges are surgery on a patient with often severely deformed asymmetric bone structures who may have been operated on many times previously with consequent presence of scar tissue. In addition, the rotational movements required commonly stretch the enveloping soft tissues to their limit. Most of the techniques are as for conventional orthognathic surgery. We will only comment here on the special considerations which have to be made for HFM cases. We will present some examples with appropriate planning.

An SLA model was produced and the existing and desired facial centre lines marked. The surgery simulated included rotational and levelling Le Fort I maxillary osteotomy, right mandibular ramus inverted L osteotomy with bone graft and left sagittal split mandibular osteotomy to advance, rotate and level the mandible and correct the occlusion to the maxilla. A rotational genioplasty advancement was also carried out. Where indicated for symmetry, onlay bone grafts were simulated. Templates and guides were prepared and all the surgery carried out in one session, bone being harvested from the anterior iliac crest.

Outcome of the rotational osteotomies and bone grafting described. He also underwent further tissue expansion to allow excision of the right pre-auricular skin-paddle which was a different colour to the surrounding skin and thus unsightly (see Figure 19h).

**Figure 19:**
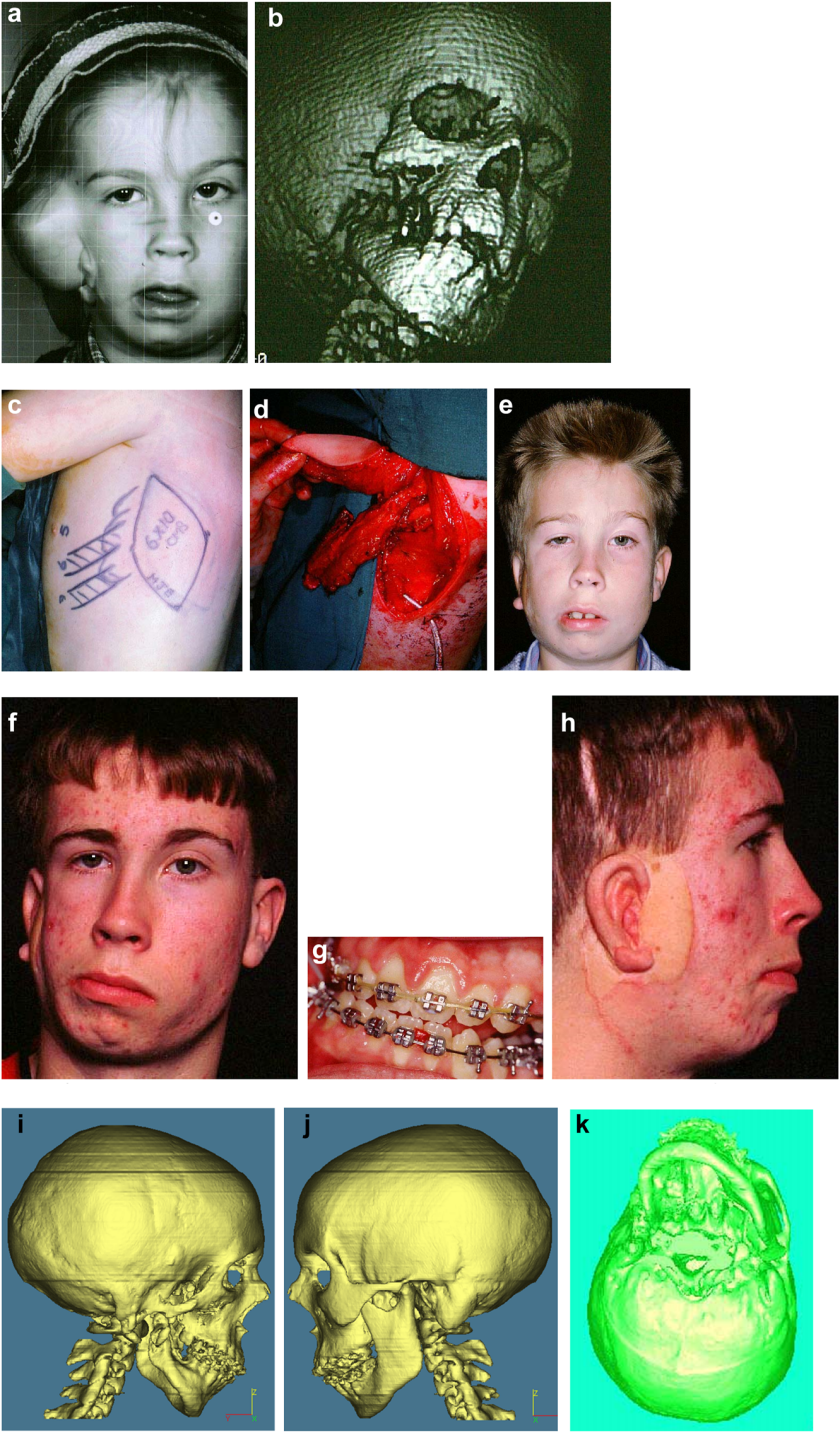
Planning for end stage reconstructrion with bimaxillary rotational osteotomies. (a and b) 8 yr old young man with PK III HFM and no zygomatic arch, glenoid fossa and mandibular condyle. Tissue expander *in situ* to produce sufficient soft tissue for ear construction. 3DCT scan at that age. (c–e) At 9 yrs of age, zygomatic arch, glenoid fossa and mand. condyle constructed with two vascularised ribs and serratus anterior muscle. (f–h) Same patient 9 years later showing established asymmetry. (i–k) Same patient’s 3D CT scan 9 years later. Significant asymmetry has developed again and pre-surgical orthodontics is completed.

**Figure 19:**
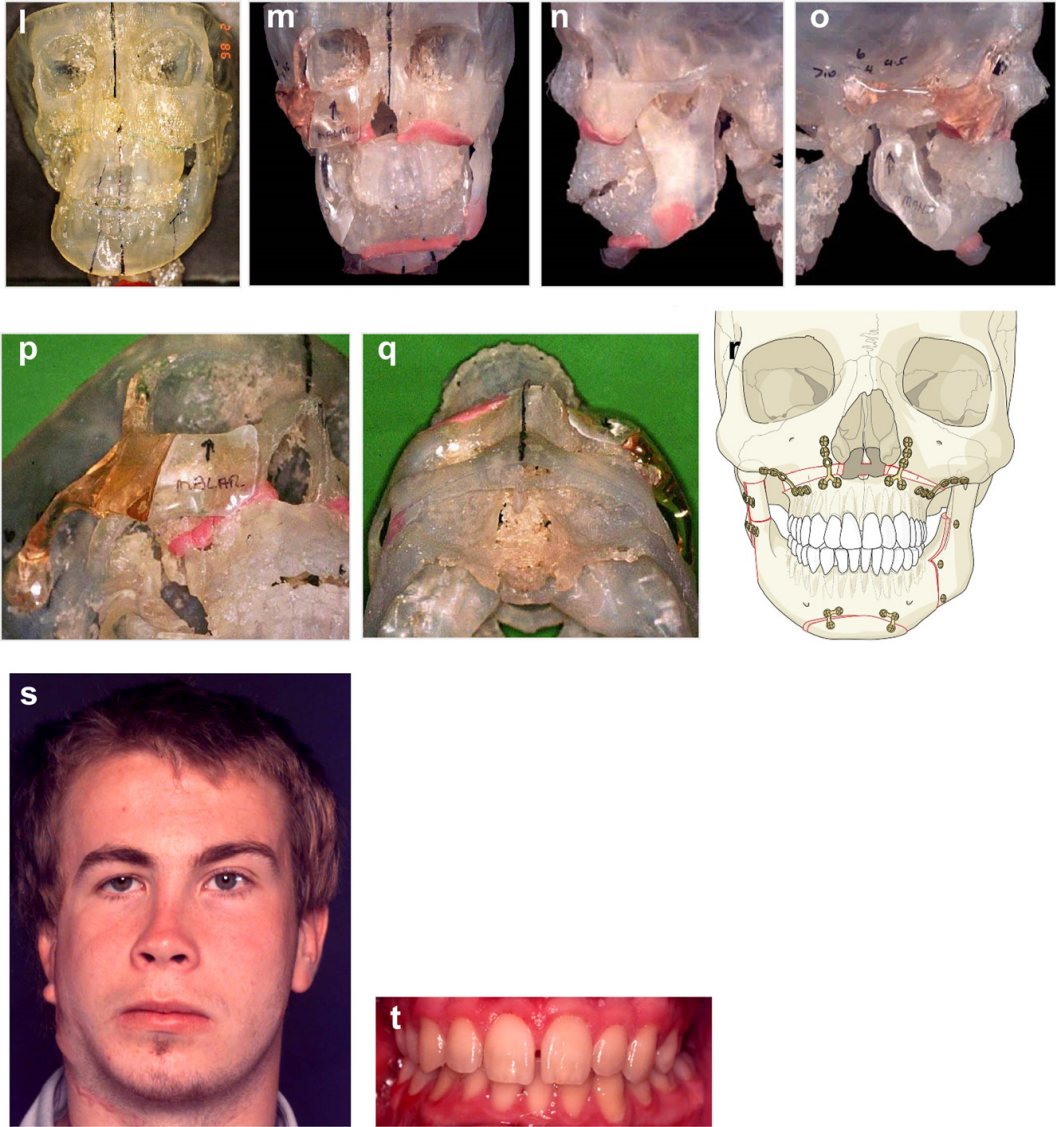
Planning for end stage reconstructrion with bimaxillary rotational osteotomies (l–t).

The further case below (Figures 20 and 21) is a PK III or IIb HFM. Please note the bone anchored hearing aid abutment *in situ*. She initially received a costo-chondral graft [34] to the left mandibular ramus.

**Figure 20:**
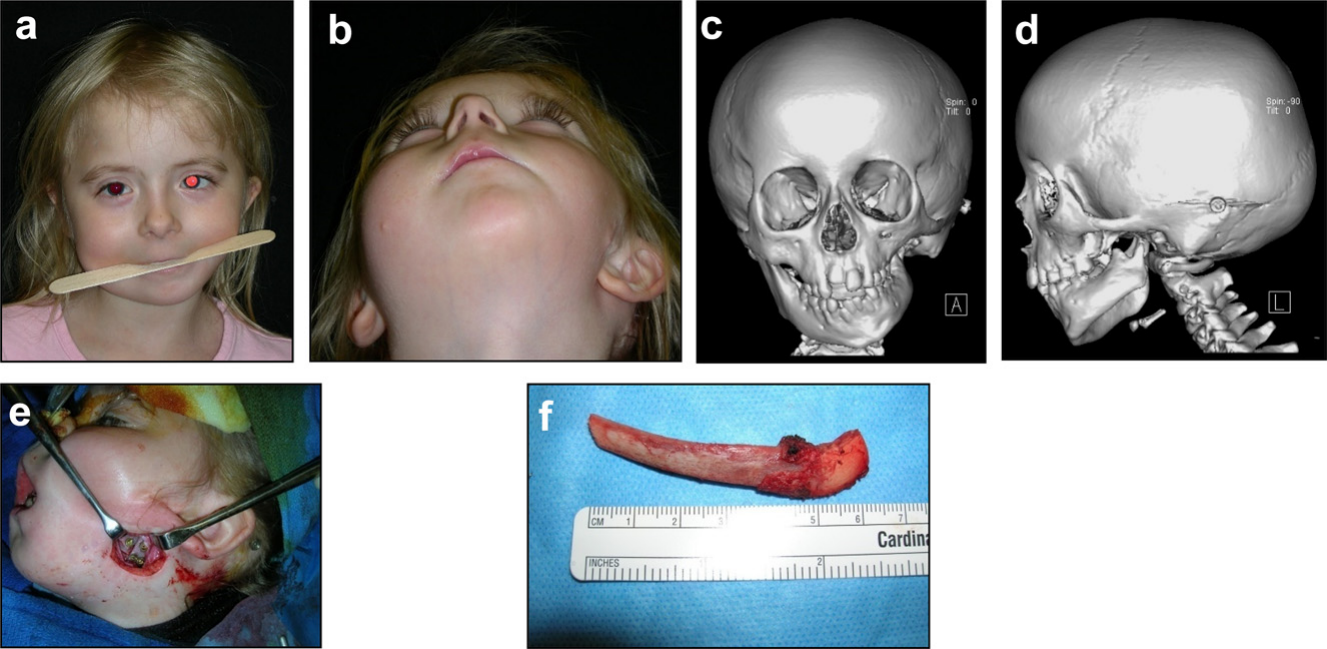
Construction of a HFM child with a costo-chondral graft followed by ramus lengthening by DO (a–f).

**Figure 20:**
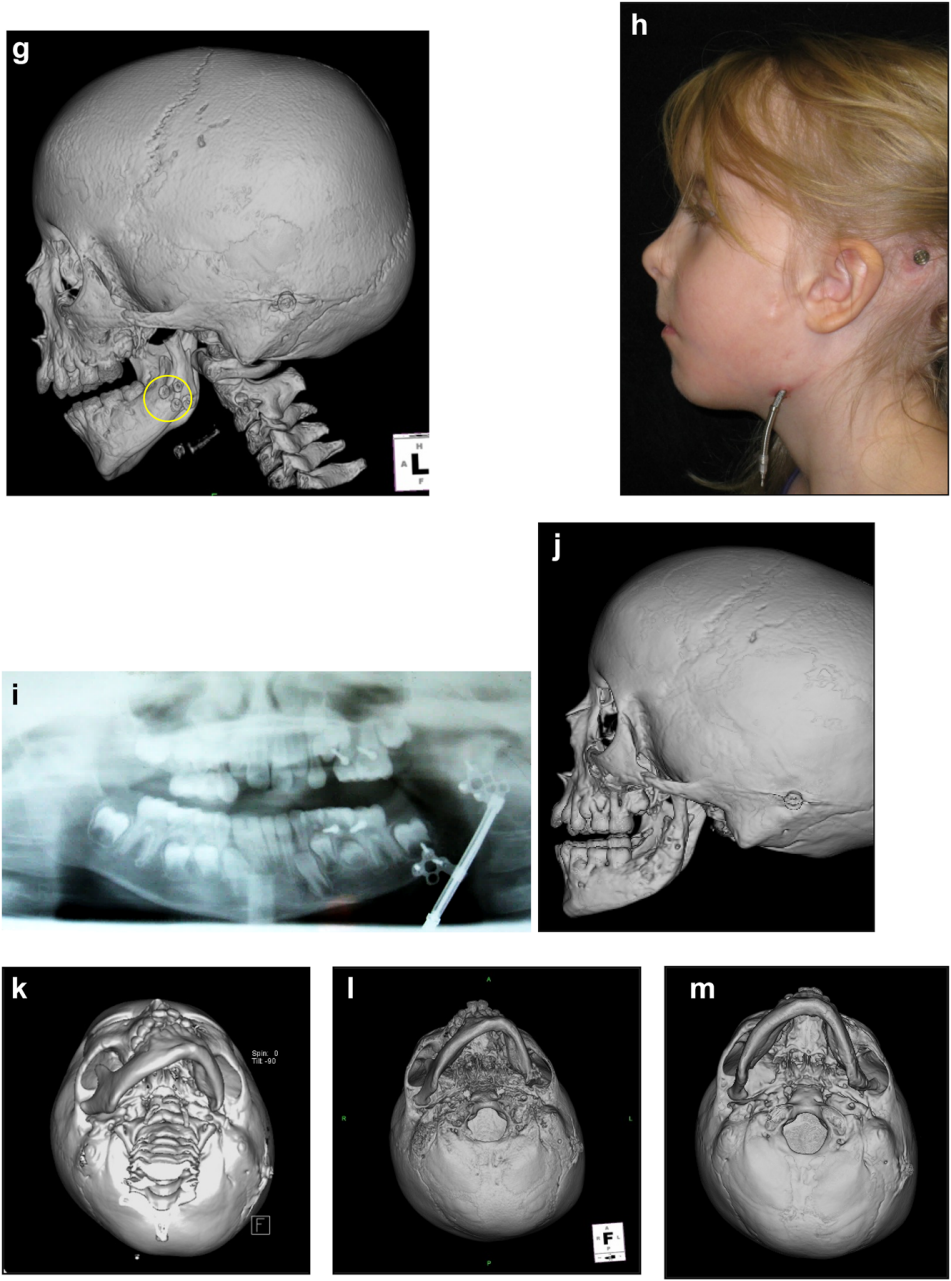
Construction of a HFM child with a costo-chondral graft followed by ramus lengthening by DO (g) Yellow ringed area is where the rib graft overlaps the original mandibular bone – the correct site for the DO osteotomy while avoiding any tooth germs. (h) Percutanous distractor port *in situ*. (i), (j), (k–m) Submental view of mandibular shape and position pre rib graft (k), post rib graft (l), post distraction (m).

Later she required vertical lenghtening of the left mandibular ramus by distraction and, as described on Page 11, it is important to perform the osteotomy for that distraction within the original mandibular bone and not in the consolidated rib graft, in the area ringed in yellow on the post-rib graft CT scan below (Figure 21).

**Figure 21:**
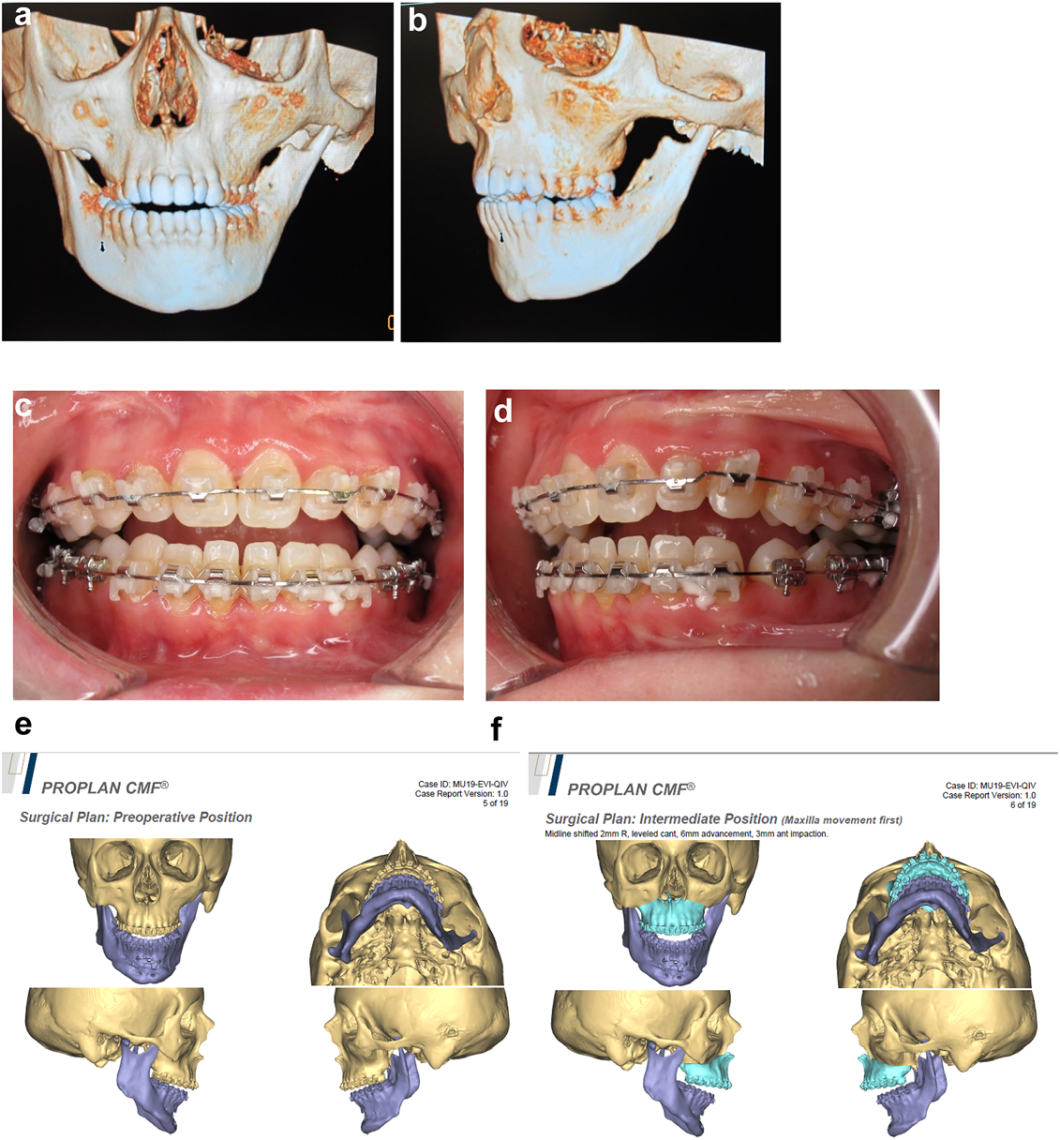
Planning of end stage reconstruction (same patient as Figure 20) by orthodontics and bimaxillary rotational osteotomies. (a and b) Same patient as Figure 20 at 15 yrs of age. (c–f) 3D pre-op and plans for the maxillary osteotomy.

**Figure 21:**
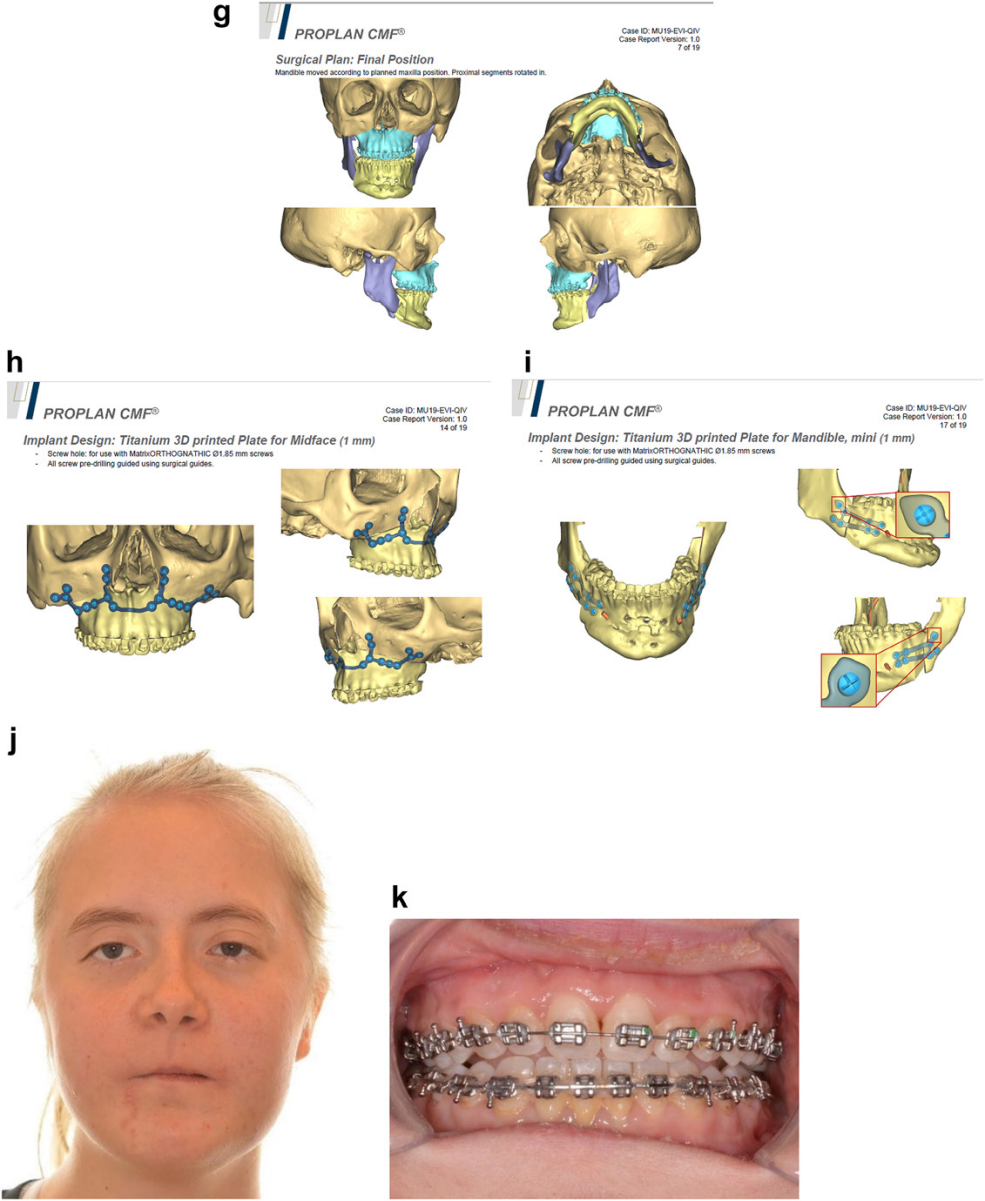
Planning of end stage reconstruction (same patient as Figure 20) by orthodontics and bimaxillary rotational osteotomies. (g) Plans for the bimax osteotomies. (h and i) Plans for printing of 3D plates for fixation of maxilla and mandible osteotomies, (j and k) Clinical outcome.

## Conclusions

In this paper we have focussed on virtual planning in the treatment of hemifacial microsomia and thus on the procedures we use or have used to treat the condition which lend themselves to such planning. There is no doubt that the use of 3D planning helps the surgeon by allowing the treatment of this complex condition by thinking through the complex requirements and trying them in the planning computer lab well in advance of surgery. By importing the patient’s own CT scans into appropriate computer software, doing the planning in 3D and then practising the procedures, each of these attempts can then be saved and if necessary changed as often as deemed necessary. This reduces some of the undoubted challenges that the surgery entails and hopefully improves the outcome for the patient. We have not used this type of planning for all of forms of surgery for this condition, either becase we did not need to or because we have not tried them to date. This paper is therefore not our comprehensive account of the treatment of HFM nor is it intended to be but we hope it demonstrates the value of such computerised 3D planning and supplements our other publications on this subject.
